# Targeting RAC1 in glioblastoma: prognostic value, immune landscape, and small molecule therapeutic potential

**DOI:** 10.3389/fonc.2026.1801747

**Published:** 2026-06-18

**Authors:** Qionghui Wu, Shanlin Chen, Xiaodong Xie, Xinli Feng, Zhenchang Zhang, Gang Su

**Affiliations:** 1 Lanzhou University Second Hospital, Lanzhou University, Lanzhou, Gansu, China; 2 Department of Neurosurgery, Liangzhou Hospital of Wuwei, Wuwei, Gansu, China; 3 Institute of Genetics, School of Basic Medical Sciences, Lanzhou University, Lanzhou, Gansu, China

**Keywords:** glioblastoma, immune infiltration, immunotherapy efficacy, lasso-Cox regression, molecular drug target, prognosis, Rac1, single-cell RNA sequencing

## Abstract

**Purpose:**

RAC1, a Rho GTPase family member involved in cell motility, is irregularly expressed in solid tumors. However, the specific tumor cell states in which RAC1 operates and its influence on the immune microenvironment remain poorly understood.

**Methods:**

RAC1 expression and prognostic value were evaluated across cancers using TCGA, HPA, CPTAC, and GEO data. After identifying GBM as the most prognostically relevant cancer type, Lasso-Cox regression and multivariate Cox analysis confirmed RAC1 as the key prognostic member among Rho GTPase family members. RAC1 expression was validated in GBM cell lines and mouse tumor tissues by qPCR and Western blotting. Effects on proliferation, migration, and invasion were assessed by CCK-8, wound healing, and Transwell assays. Immune infiltration, immunotherapy responsiveness, and drug sensitivity were analyzed using TCGA data. Single-cell RNA sequencing analysis was performed to resolve RAC1 expression across tumor cell states and the immune microenvironment. Molecular docking and *in vitro* CCK-8 assays were used to evaluate Palbociclib as a candidate drug.

**Results:**

RAC1 was upregulated in most cancers, with the highest prognostic accuracy in GBM. Lasso-Cox regression retained RAC1 across OS, DSS, and PFI endpoints, and multivariate Cox analysis confirmed it as the sole family member with HR exceeding 2 (HR = 2.61, P = 0.003). NSC23766 suppressed GBM cell proliferation, migration, and invasion without altering total RAC1 protein, confirming dependence on RAC1 activation state. Single-cell analysis revealed RAC1 enrichment in the NPC-like tumor state, independent of cell cycle. RAC1-high cells exhibited PI3K-AKT-mTOR and IFN-gamma pathway activation with upregulated MHC-I but absent PD-L1, explaining ICI resistance. Palbociclib inhibited GBM cell viability in a dose- and time-dependent manner.

**Conclusion:**

RAC1 serves as a prognostic biomarker and therapeutic stratification indicator in GBM, marking IFN-responsive tumor subpopulations that may benefit from CDK4/6 inhibitor-based strategies rather than ICI monotherapy.

## Graphical Abstract

**Figure:**
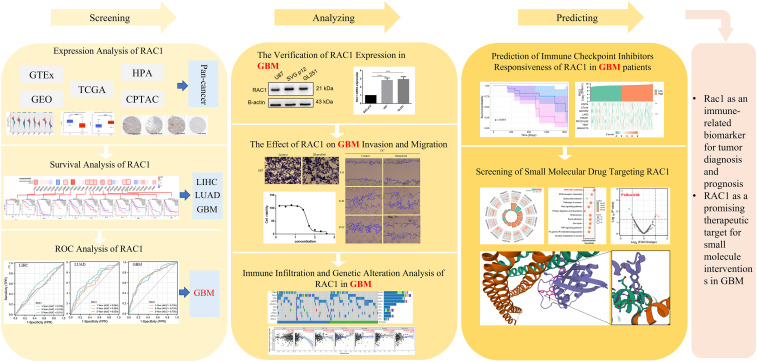

## 1 Introduction

RAC1, a highly significant and conserved member of the RHO family (1–3), plays a crucial role in mediating changes in cell behavior by interacting with various effector proteins (4–7), which is regulated by Rho guanine nucleotide exchange factors (8). RAC1 is expressed extensively in tissues and is involved in cell movement and invasion (9). However, some solid tumors have shown a correlation between tumorigenesis and RAC1 expression level (10–15). Studies have demonstrated that the dysregulation of RAC1 expression and regulation contributed to tumorigenesis through various mechanisms, including epithelial-mesenchymal transition, cell proliferation cycle, migration, invasion, drug resistance, and pro-angiogenic (1, 15, 16). However, the different tumor-promoting effects of RAC1 in different types of tumors are not yet clear, so we analyzed the expression differences and prognostic effects of RAC1 in different tumors based on the TCGA database, speculating that RAC1 may have better predictive ability and potential tumor-promoting effect in glioblastoma.

Glioblastoma (GBM), the most prevalent and deadly primary intracranial tumor, remains a significant challenge despite advancements in surgery and adjuvant therapy. The median overall survival of patients with GBM still does not exceed 15 months (17). Therefore, it is imperative to uncover the molecular mechanisms of GBM and develop novel therapeutic strategies. The utilization of immune checkpoint inhibitors in immunotherapy has substantially enhanced the objective response rate and overall survival of patients with advanced malignant tumors. However, the overall effective rate of a single drug was less than 20%, and the cost was generally high (18). Therefore, it is urgent to find accurate and reliable biomarkers to screen potential benefit patients of immunotherapy, so that different treatment modalities can be used more selectively.

Recent advances in single-cell RNA sequencing (scRNA-seq) have revealed that GBM tumors harbor four distinct malignant cell states: mesenchymal-like (MES), neural progenitor cell-like (NPC), oligodendrocyte progenitor cell-like (OPC), and astrocyte-like (AC) (19). Understanding which cell states drive therapeutic resistance and immune evasion is critical for developing effective treatment strategies. However, the specific cell states in which RAC1 operates and its role in the tumor microenvironment remain poorly characterized at single-cell resolution.

In this study, we first evaluated the expression and prognosis of RAC1 in various tumors based on TCGA, supplemented by CPTAC proteomic data. After identifying GBM as the cancer type with the strongest prognostic relevance for RAC1, we performed Lasso-Cox regression and multivariate Cox analysis to establish a data-driven rationale for focusing on RAC1 among all Rho GTPase family members. Accordingly, we validated the expression of RAC1 in GBM and explored its impact on the proliferation, migration, and invasion of GBM cells. Additionally, we analyzed the mutational status of the RAC1 gene within GBM and its correlation with immune infiltration. We further integrated public scRNA-seq data to resolve RAC1 expression across distinct tumor cell states and immune cell populations, elucidating the cellular mechanisms underlying immunotherapy resistance. Finally, we predicted the response of abnormal expression of RAC1 to immune checkpoint therapy such as PD-1 antibody. In order to screen small molecular drugs that may target RAC1, the key pathways of RAC1 participating in GBM was analyzed, providing reference for finding effector molecules of RAC1 and potential therapeutic schemes in GBM.

## 2 Materials and methods

### 2.1 Gene expression analysis and prognosis analysis

TIMER2 (20) and GEPIA2.0 (**Table 1**) (21) drew a comparison between RAC1 expression in tumors and normal tissues based on TCGA and Genotype-Tissue Expression (GTEx) data. UALCAN (22) was used to examine RAC1 protein expression in the Clinical Proteomic Tumor Analysis Consortium (CPTAC). To provide comprehensive proteomic-level evidence, we additionally incorporated CPTAC pan-cancer proteomic data processed by LinkedOmicsKB, extracting RAC1 protein abundance from ten CPTAC tumor cohorts. For GBM-specific analysis, CPTAC-GBM phosphoproteomic data were analyzed to identify detectable RAC1 phosphosites and their correlation with total protein abundance. The protein expression data of RAC1 in different tumors were obtained from the Human Protein Atlas (HPA http://www.proteinatlas.org/). Of note, the HPA Pathology database classifies brain tumors under the general category of “glioma” without a separate GBM entry; the glioma IHC results shown include GBM specimens. The expression of RAC1 in LUAD, LIHC, and GBM was confirmed using GEO dataset. The R software was utilized for further visualization and statistical analysis. GEPIA2.0 was used to acquire overall survival (OS), disease-free survival (DFS) data and survival curves of RAC1 in TCGA. The prognostic information was verified by GEO dataset and Kaplan-Meier Plotter. The “Forest plot” R package was utilized for statistical analysis.

**Table 1:** Primer sequence.

Primer	Sequence
RAC1-FP	GAGACGGAGCTGTTGGTAAAA
RAC1-RP	ATAGGCCCAGATTCACTGGTT
ACTIN-FP	GTTGGACCTGACAGACTACCTCA
ACTIN-RP	GTTGCCAATAGTGATGACCT

### 2.2 Lasso-Cox regression and multivariate Cox analysis

To provide an unbiased rationale for prioritizing RAC1 among Rho GTPase family members, Lasso-penalized Cox proportional hazards regression was performed using the glmnet R package (v4.1) on TCGA-GBM RNA-seq data. All 18 detectable Rho GTPase family members (RAC1, RAC2, RAC3, RHOA, RHOB, RHOC, CDC42, RND1, RND2, RND3, RHOG, RHOF, RHOD, RHOQ, RHOU, RHOV, RHOT1, RHOT2) were included as candidate predictors. Ten-fold cross-validation was used to select the optimal regularization parameter. Lasso-Cox was performed separately for OS, DSS, and PFI endpoints. Genes selected by Lasso (non-zero coefficients) were subsequently entered into a multivariate Cox proportional hazards model to determine independent prognostic significance, with hazard ratios and 95% confidence intervals reported.

### 2.3 Cell line culture

Human glioma U87 and U251 cell lines were obtained from the Cell Resource Center, Peking Union Medical College. Cell line identities were confirmed by STR profiling (**Supplementary Figure 1**), and mycoplasma testing was negative. Human astrocytes SVG p12 were obtained from ShangEn Biosciences (Wuhan, China). Cell lines were cultured by DMEM medium (BasalMedia, China, Shanghai) supplemented with 10% fetal bovine serum (Gibco; Thermo Fisher Scientific, Inc.) at 37 degrees C in 5% CO2.

### 2.4 Cell counting kit-8 assay

Cells were seeded at a density of 2x10^4 cells/mL in a 96-well plate (100 uL per well) and incubated with varying concentrations of RAC1 inhibitor NSC23766(Cat. No. HY-15723, MedChemExpress) or Palbociclib (Cat. No. HY-50767, MedChemExpress) at 37 degrees C in an atmosphere of 5% CO2 for either 24 or 48 hours. Subsequently, 10 uL of CCK-8 solution (Catalog No. K1018, APExBIO, USA) was added to each well. Cell viability was then estimated by measuring the absorbance at 450 nm using a microplate reader (Synergy NEO2, Agilent, USA).

### 2.5 Wound healing assay

Cells were evenly seeded in six-well plates, and once they reached 90% confluence, a single layer of cells was scraped with a pipette tip to create a wound in the culture dish. The cells were then incubated for 48 hours in serum-free medium to eliminate the influence of cell proliferation. Wound images were captured at 0, 24, and 48 hours using an optical microscope (ECLIPSE NI, Nikon, Japan). Wound healing was quantified by measuring the remaining wound area at each time point using ImageJ software.

### 2.6 Transwell assay

Cells (1x10^5) in serum-free medium were seeded onto the upper chamber of Matrigel-coated (1:6; BD, USA) Transwell inserts (8 um pore size, Corning Costar, USA), while the lower chamber was filled with medium containing 20% serum. The cells were cultured for 48 hours in a humidified incubator at 37 degrees C with 5% CO2. Subsequently, cells that had invaded to the bottom surface of the Transwell were stained with 0.5% crystal violet and counted under an inverted microscope (IX83, OLYMPUS, Japan). All experiments were performed in triplicate.

### 2.7 Quantitative real-time PCR

Total RNA content of tissues and cells was extracted using TRIzol reagent (Takara). Reverse transcription using PrimeScript kit (Takara). The relative expression of RAC1 was detected using SYBR Green PCR kit (Takara, Japan) based on the 2^-ddCt method and normalized with ACTIN.

### 2.8 Western blot assay

Protein samples were separated by 8-12% sodium dodecyl sulfate-polyacrylamide gel electrophoresis (SDS-PAGE) and transferred to a PVDF membrane (Millipore, USA). After blocking with 5% BSA, the membrane was incubated overnight at 4 degrees C with the appropriate primary antibody anti-RAC1 (1:600; Proteintech, China), followed by incubation with the anti-rabbit IgG (1:3,000; Proteintech, China) at room temperature for 1 hour. Membrane visualization was performed using the chemiluminescent (ECL) Western Blotting Substrate (NCM Biotech, China), the FUSION SOLO6S system (VILBER, France).

### 2.9 Genetic alteration analysis, immune infiltration and immunotherapy response analysis

Based on cBioPortal (23, 24), we acquired data on RAC1 gene alterations from TCGA. To describe the correlation between quantitative variables without a normal distribution, we employed Spearman’s correlation analysis. The immune infiltration was explored by TIMER2. The differential expression of RAC1 in GBM patients with non-response and response to PD-1 treatment and prognosis of patients with RAC1 differential expression after PD-1 treatment [Dataset ID: GBM-PRJNA482620_anti-PD-1 (25)] was obtained based on TIGER. The TIDE (26) algorithm was used to predict response to immune checkpoint inhibitors.

### 2.10 RAC1-related gene enrichment analysis

After obtaining the first 100 RAC1-related genes in TCGA, protein-protein interaction networks were created using STRING (https://string-db.org/). Jvenn (27) was used to perform a Venn analysis on RAC1-related genes and up-regulated genes in GBM. Additionally, we conducted GO analysis and KEGG pathway analysis using Metascape (28).

### 2.11 Drug sensitivity analysis

The Genomics of Drug Sensitivity in Cancer (GDSC) was employed to obtain drug IC50 values and predict the chemotherapeutic response of each sample. The prediction procedure was carried out using the “prophetic” R software package. To evaluate the prediction accuracy based on the GDSC training set, tenfold cross-validation was employed (29).

### 2.12 Molecular docking

AutodockVina1.2.2 (30) was utilized to examine the binding affinities and interaction modes between the drug candidate and their targets. The molecular structure of Palbociclib was obtained from PubChem Compound (31). The 3D coordinate of Rac1 was downloaded from the PDB. All protein and molecular files were converted into PDBQT format for docking analysis, which was performed using AutodockVina.

### 2.13 Single-cell RNA sequencing analysis

Publicly available GBM single-cell RNA sequencing data were obtained from the Gene Expression Omnibus (GSE131928; Neftel et al., Cell, 2019) (19). The 10X Chromium dataset was used for all analyses. Raw count matrices were processed using the Seurat R package (v5.0). Quality control was performed by retaining cells with 500-7,500 detected genes, fewer than 50,000 UMI counts, and mitochondrial gene content below 15%. After filtering, data were log-normalized with a scale factor of 10,000, and the top 3,000 highly variable genes were identified using the variance-stabilizing transformation method. Expression values were scaled with regression of mitochondrial gene percentage and UMI counts. Principal component analysis was performed, and the number of significant PCs was determined based on the inflection point of the ElbowPlot. UMAP dimensionality reduction and Louvain clustering were subsequently applied, with clustering resolution evaluated across multiple values to optimize marker gene specificity.

Cell type and malignant cell state annotations (MES-like, NPC-like, OPC-like, and AC-like) were obtained directly from the original publication metadata. For multi-patient data integration, Harmony batch correction was applied prior to downstream analysis to mitigate patient-specific technical variation.

Within the malignant cell compartment, AUCell (32) was used to calculate pathway activity scores based on the Neftel-defined gene signatures for each of the four tumor cell states. Each malignant cell was assigned a dominant state based on its highest AUCell score. Cell cycle phase assignment was performed using the CellCycleScoring function in Seurat with S-phase and G2M-phase gene sets defined by Tirosh et al. (33). To assess whether the association between RAC1 expression and NPC-like state was independent of cell cycle effects, expression data were re-scaled with regression of S.Score and G2M.Score, and Spearman correlations were recalculated on the resulting residuals.

Differential gene expression between RAC1-high and RAC1-low tumor cells, defined by median expression split, was performed using the Wilcoxon rank-sum test via the FindMarkers function. Gene Set Enrichment Analysis was conducted using clusterProfiler (34) with the MSigDB Hallmark and Reactome gene set collections. Single-cell co-expression of RAC1 and CDK4 was evaluated by Spearman correlation and visualized using dual-gene FeaturePlot. Expression of IFN-gamma signaling components and immune checkpoint molecules (JAK1, JAK2, STAT1, IRF1, CD274, PDCD1LG2, B2M, HLA-A, HLA-B, HLA-C) was compared between RAC1-high and RAC1-low groups using DotPlot visualization.

### 2.14 Statistical analyses

Statistical analyses were conducted using SPSS 22.0 software, GraphPad Prism 6.0, and R (v4.3). All experiments were independently replicated three times, and all quantitative analyses are presented as the mean +/- standard deviation (SD). Independent t-tests were applied to compare two groups. Spearman’s correlation analysis was employed to assess the significance of associations between quantitative variables. For single-cell analyses, Wilcoxon rank-sum tests were used for group comparisons, and Kruskal-Wallis tests for multi-group comparisons.

## 3 Results

### 3.1 The expression analysis of RAC1 across cancers

**Figure 1:**
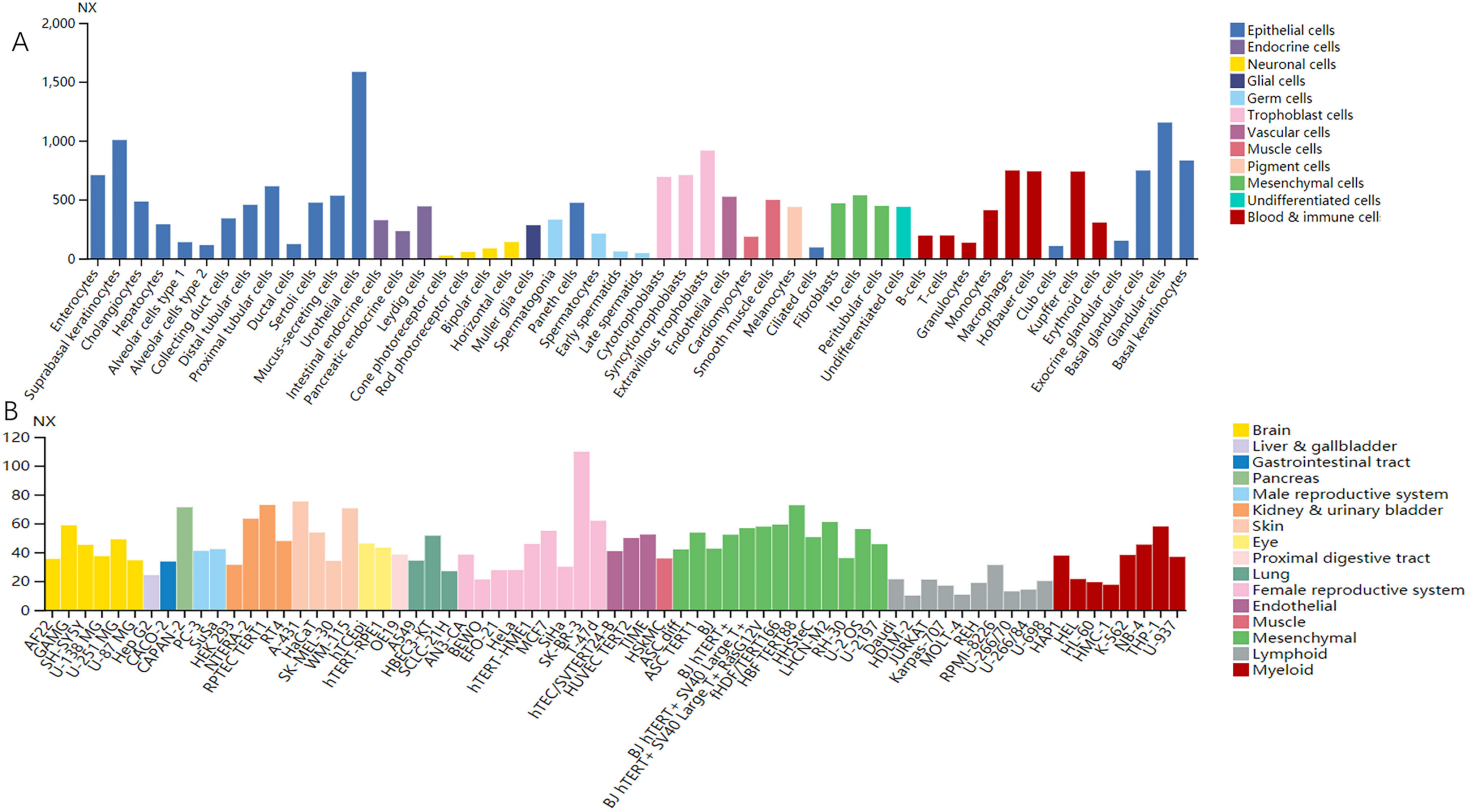
Expression level of RAC1 in different cells and tissues under normal physiological conditions. **(A)** Expression of RAC1 gene in different tissues based on HPA; **(B)** Expression of RAC1 gene in cell lines based on the HPA.

**Figure 2:**
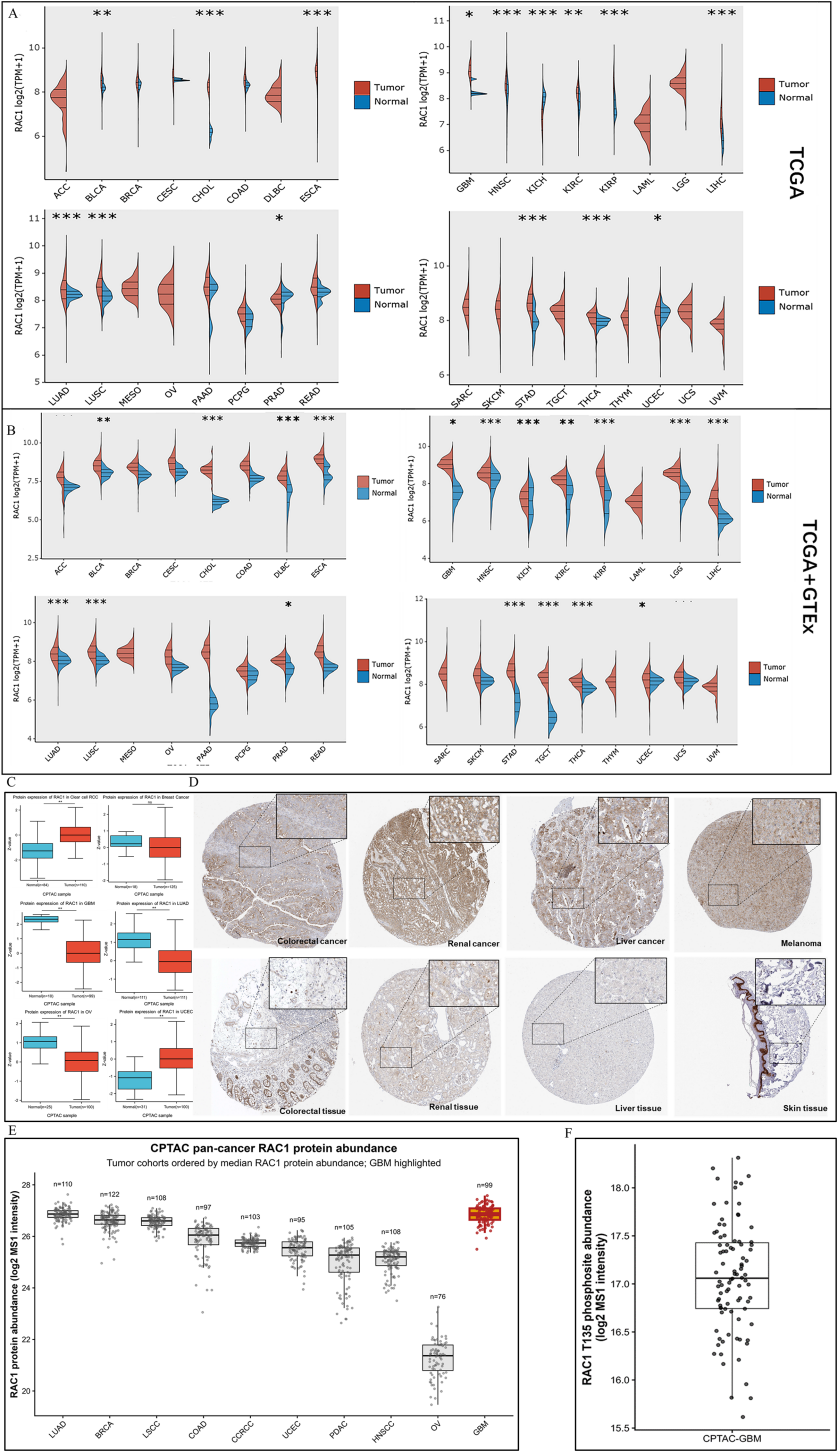
The expression of RAC1 in tumor tissues and normal tissues. **(A)** The gene expression of RAC1 in the TCGA database; **(B)** The gene expression of RAC1 in TCGA database combined with the GTEx database; **(C)** The protein expression of RAC1 based on CPTAC dataset; **(D)** The relative abundance of RAC1 protein expression based on HPA cohort; **(E)** CPTAC pan-cancer distribution of RAC1 protein abundance; **(F)** CPTAC-GBM RAC1 T135 phosphosite analysis. (* *P* < 0.05, ** *P* < 0.01, *** *P* < 0.001)

Based on the HPA database, the expression of RAC1 was generally high in epithelial cells, especially in urothelial cells (**Figure 1A**). Pancreas-related cell lines commonly exhibit higher expression of RAC1 compared to other cell lines (**Figure 1B**). TCGA (**Figure 2A**) and GTEx data (**Figure 2B**) revealed that RAC1 was highly expressed in multiple tumor tissues (BLCA, CHOL, ESCA, GBM, HNSC, KICH, KIRC, KIRP, LIHC, LUAD, LUSC, STAD, THCA, PRAD, UCEC, DLBC, LGG and TGCT) (**Supplementary Table 1**) compared to normal tissues. We further conducted a protein expression study of RAC1 in various tumors using CPTAC and HPA as our basis. Based on the CPTAC dataset (**Figure 2C**), OV, LUAD, KIRC, and UCEC had higher protein expression levels of RAC1 compared to normal tissues, while GBM had lower protein expression levels. The HPA cohort (**Figure 2D**) showed that liver cancer, renal cancer, and melanoma had higher RAC1 expression levels compared to normal tissues. In summary, the transcription and protein expression levels of RAC1 in KIRC, LIHC, LUAD, and GBM were different from those in normal tissues.

To provide a comprehensive proteomic landscape, we additionally analyzed CPTAC pan-cancer proteomic data from LinkedOmicsKB. RAC1 protein abundance was detectable across all ten CPTAC tumor cohorts, with GBM (n = 99) showing relatively high protein abundance (**Figure 2E**). Furthermore, CPTAC-GBM phosphoproteomic analysis identified the RAC1 T135 phosphosite in 90 GBM samples. The raw phosphosite abundance showed a modest positive correlation with total RAC1 protein abundance (Spearman rho = 0.258, P = 0.0142). However, after correcting for total protein abundance, the adjusted T135 phosphorylation index showed a negative correlation with RAC1 total protein (Spearman rho = -0.274, P = 0.0091), and RAC1-high tumors exhibited lower relative T135 phosphorylation than RAC1-low tumors (Wilcoxon P = 0.00922; **Figure 2F**). Since RAC1 functional activation is primarily determined by GTP-binding status rather than T135 phosphorylation, these results were not interpreted as evidence of differential RAC1 activation.

### 3.2 Pan-cancer survival analysis identifies GBM as the cancer type with the strongest prognostic relevance for RAC1

**Figure 3:**
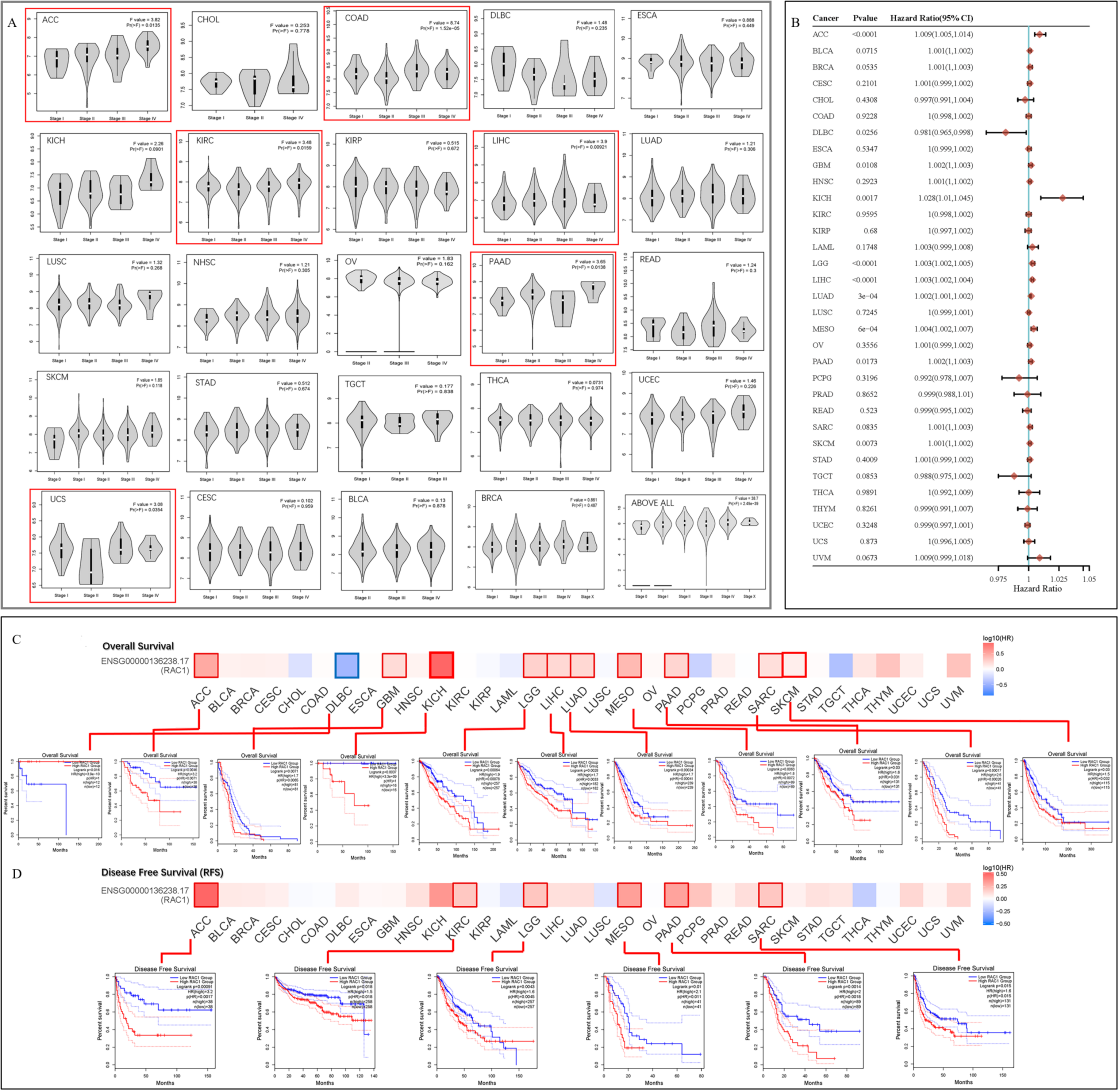
Pan-cancer survival analysis of RAC1. **(A)** RAC1 expression at different pathological stages across TCGA tumors; **(B)** Forest plot of RAC1 expression and overall survival across cancer types; **(C)** OS survival curves of RAC1 in selected tumors using GEPIA2.0; **(D)** DFS survival curves of RAC1 in selected tumors.

Using GEPIA2.0, we analyzed the RAC1 expression at different pathological stages of all tumors in TCGA (**Figure 3A**). In ACC, COAD, LIHC, KIRC, PAAD, and UCS, RAC1 expression was found to be correlated with tumor stage. In contrast, no significant correlation was found between RAC1 expression and tumor stage in other types of tumors. Given that tumor stage is believed to have a direct impact on patient prognosis, we examined the correlation between RAC1 expression and OS prognosis in different tumor types using TCGA data (**Figure 3B**). Our findings revealed that over-expression of RAC1 was associated with poor OS in patients with ACC, GBM, LGG, LIHC, LUAD, MESO, PAAD, SARC, SKCM, and KICH. In contrast, high expression of RAC1 was found to be a protective factor in DLBC. We further analyzed the prognosis using GEPIA2.0. The OS prognosis data (**Figure 3C**) were consistent. The DFS data (**Figure 3D**) showed that elevated expression of RAC1 in ACC, KIRC, LGG, MESO, PAAD, and SARC was associated with shortened DFS. In summary, RAC1 was not only differentially expressed in GBM, LIHC, and LUAD but also related to OS.

**Figure 4:**
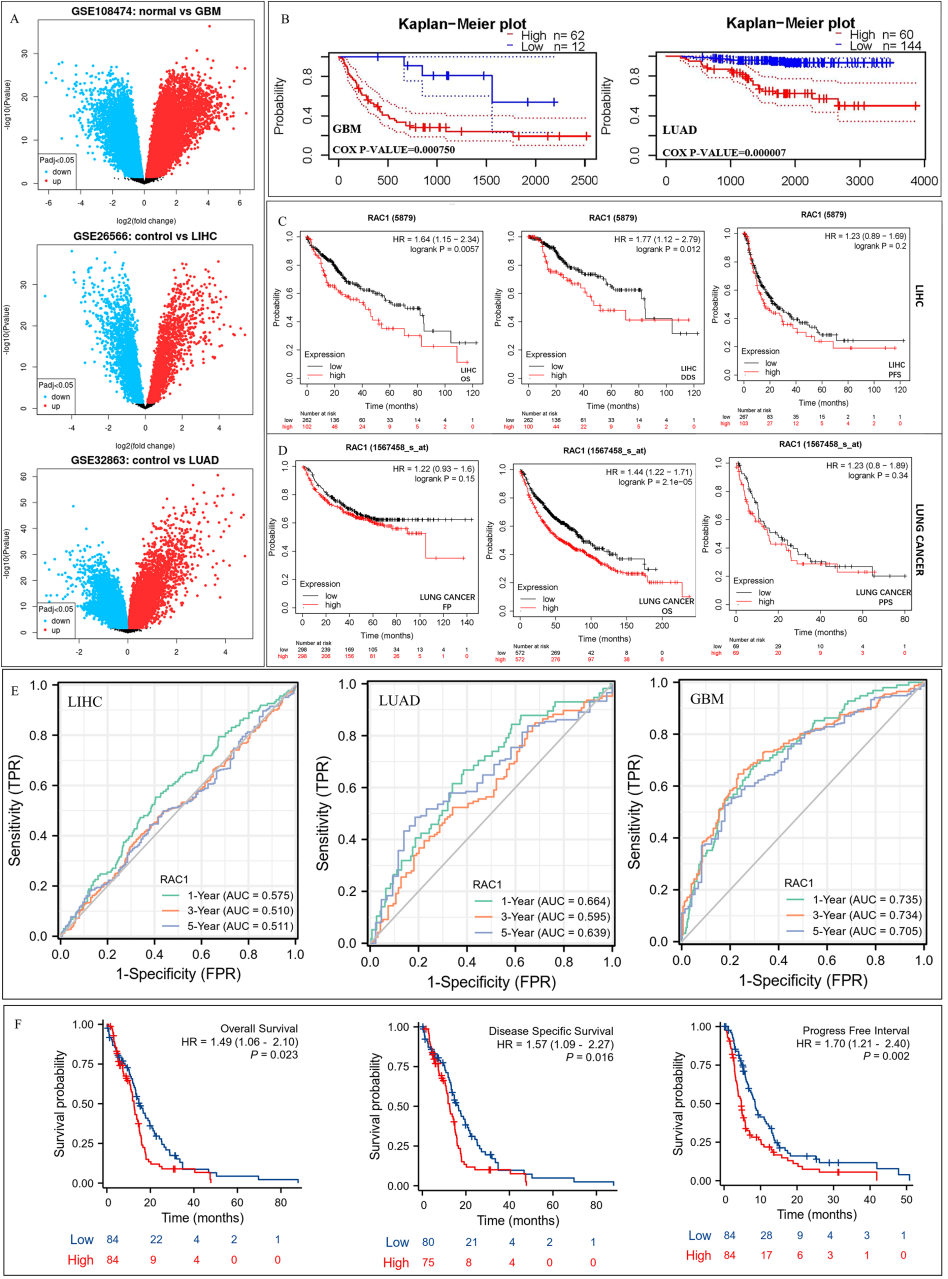
Validation of RAC1 expression and prognosis. **(A)** Analysis of differential genes in GBM, LIHC, LUAD based on GEO; **(B)** Analysis of the relationship between the high expression of RAC1 and overall survival in GBM and LUAD based on GEO; **(C)** The relationship between RAC1 expression and OS, DDS, PFS in LIHC; **(D)** The relationship between RAC1 expression and FP, OS and PPS in lung cancer; **(E)** ROC curve analysis of prognosis in GBM, LIHC, and LUAD; **(F)** GBM-specific OS, DSS, PFS Kaplan-Meier curve.

To further investigate, we analyzed RAC1 expression in GBM (GSE108474), LIHC (GSE26566), and LUAD (GSE32863) using the GEO dataset (**Figure 4A**). RAC1 expression was up-regulated in GBM, LIHC, and LUAD compared to normal tissues. The analysis revealed that high RAC1 expression in GBM (GSE4412) and LUAD (GSE31210) was associated with poor OS (**Figure 4B**). We combined the Kaplan-Meier data to analyze the correlation between RAC1 expression and OS, DDS, PFS, first progression, and post-progression survival in LIHC (**Figure 4C**) and lung cancer (**Figure 4D**). We utilized the ROC curve to evaluate the prognostic ability of RAC1. The AUC scores for 1-year OS in GBM, LIHC, and LUAD were 0.735, 0.575, and 0.664, respectively. For 3-year OS, the AUC scores were 0.734, 0.510, and 0.595, respectively. For 5-year OS, the AUC scores were 0.705, 0.511, and 0.639, respectively (**Figure 4E**). These results indicate that RAC1 shows the highest prognostic accuracy in GBM across all time points. As a result, subsequent analyses were focused on abnormal RAC1 expression in GBM.

Additionally, GBM-specific survival analysis using TCGA-GBM clinical data confirmed that high RAC1 expression was significantly associated with poor OS, DSS and shorter PFS (**Figure 4F**) in GBM patients, further supporting the prognostic value of RAC1 specifically in GBM.

### 3.3 RAC1 is the sole independent prognostic factor among Rho GTPase family members in GBM

**Figure 5:**
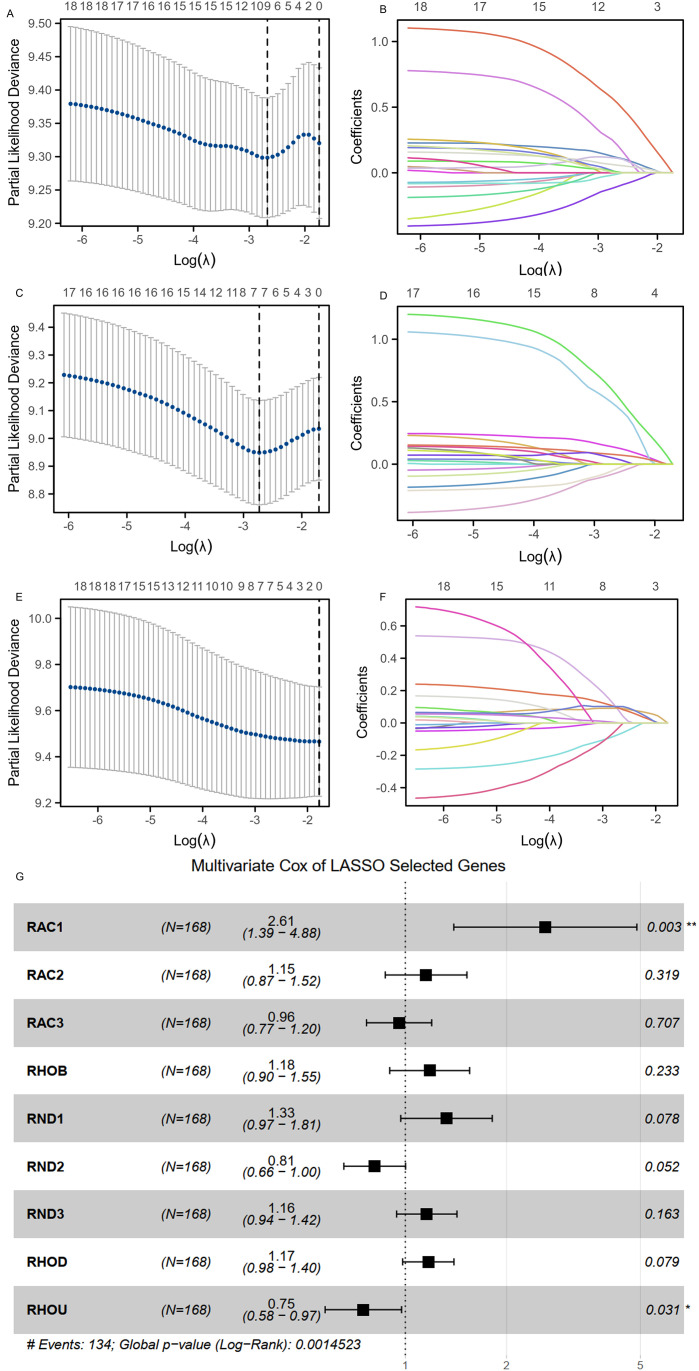
RAC1 is the sole independent prognostic factor among Rho GTPase family members. **(A, B)** Lasso-Cox cross-validation curve and coefficient path for OS; **(C, D)** Lasso-Cox cross-validation curve and coefficient path for DSS; **(E, F)** Lasso-Cox cross-validation curve and coefficient path for PFI; **(G)** Multivariate Cox regression forest plot of Lasso-selected genes (RAC1: HR = 2.61, P = 0.003).

Having established GBM as the cancer type where RAC1 exhibits the strongest prognostic relevance (Sections 3.1-3.2), we next sought to determine whether RAC1 should be prioritized over other Rho GTPase family members. To address this question, Lasso-Cox regression was performed on TCGA-GBM data using all 18 detectable Rho GTPase family members as candidate predictors. Ten-fold cross-validated Lasso-Cox analysis was conducted separately for three clinical endpoints: overall survival (OS), disease-specific survival (DSS), and progression-free interval (PFI). RAC1 was consistently retained with a non-zero coefficient across all three endpoints (**Figures 5A–F**), demonstrating robust prognostic relevance regardless of the survival metric used.

To further confirm the independent prognostic value of the Lasso-selected genes, multivariate Cox proportional hazards analysis was performed. Among the nine genes selected by Lasso, RAC1 exhibited the highest hazard ratio (HR = 2.61, 95% CI: 1.39-4.88, P = 0.003; **Figure 5G**), indicating that each unit increase in RAC1 expression was associated with a 2.61-fold increase in mortality risk. Notably, RAC1 was the only family member with both a hazard ratio exceeding 2 and a P-value below 0.01. While RHOU also reached statistical significance (HR = 0.75, P = 0.031), it exhibited a protective rather than oncogenic effect. The remaining seven Lasso-selected genes (RAC2, RAC3, RHOB, RND1, RND2, RND3, RHOD) did not achieve significance in multivariate analysis (all P > 0.05). These results establish RAC1 as the most significant independent prognostic risk factor within the entire Rho GTPase family in GBM.

### 3.4 Aberrant expression of RAC1 in GBM promotes proliferation, migration, and invasion

**Figure 6:**
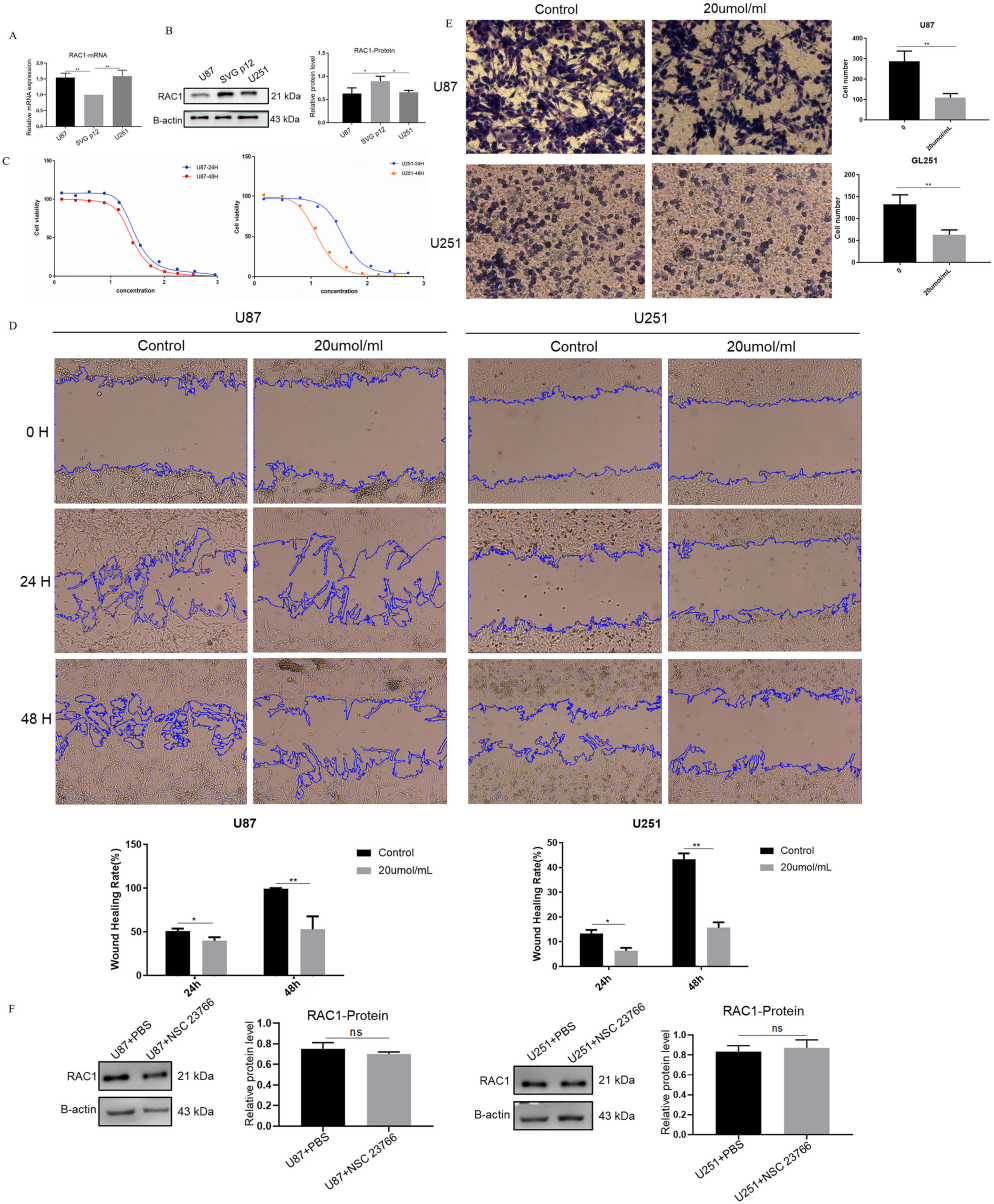
Effect of RAC1 on GBM proliferation, migration, and invasion. **(A)** mRNA expression levels of RAC1 in SVG p12, U87, and U251 cell lines. **(B)** Protein expression levels of RAC1 in SVG p12, U87, and U251 cell lines. **(C)** Proliferation of U87 and U251 cells treated with varying concentrations of NSC23766, as assessed by CCK-8 assay. **(D)** Migration of U87 and U251 cells treated with 20 uM NSC23766, as evaluated by wound healing assay. **(E)** Invasion of U87 and U251 cells treated with 20 uM NSC23766, as determined by Transwell assay. **(F)** Western blot analysis of RAC1 protein expression in U87 and U251 cells after NSC23766 treatment, with quantification ((*P < 0.05, **P < 0.01) ns, not significant).

To assess the expression of RAC1 in GBM, we extracted mRNA from human astrocyte SVG p12 cells, human glioblastoma cell lines U87 and U251, tumor tissue from a mouse glioma orthotopic model, and normal mouse brain tissue. qPCR (**Figure 6A**) and Western blotting (**Figure 6B**) results revealed that, compared to human astrocyte SVG p12 cells, RAC1 mRNA was upregulated but protein expression was downregulated in U87 cells. Similarly, in the mouse glioma orthotopic model, RAC1 mRNA expression was elevated, while protein levels were reduced in tumor tissue compared to normal brain tissue. To investigate the impact of RAC1 on GBM proliferation, U87 and U251 cells were treated with different concentrations of the RAC1 inhibitor NSC23766. CCK-8 assays (**Figure 6C**) indicated that the half-maximal inhibitory concentrations (IC50) for U87 cells at 24 and 48 hours were 46 uM and 30 uM, respectively, and for U251 cells, they were 52 uM and 36 uM, showing a concentration-dependent effect. Furthermore, treatment with 20 uM NSC23766 significantly inhibited the migration and invasion of both U87 and U251 cells, as demonstrated by wound healing (**Figure 6D**) and Transwell assay (**Figure 6E**).

To verify the effect of NSC23766 on RAC1 protein expression, we examined RAC1 protein levels by Western blotting after NSC23766 treatment (**Figure 6F**). Total RAC1 protein expression was not significantly altered in either U87 or U251 cells following NSC23766 treatment compared to PBS controls. This result is consistent with the known mechanism of NSC23766, which functions as a RAC1-specific GEF inhibitor that blocks the interaction between RAC1 and its activating guanine nucleotide exchange factors (particularly Trio and Tiam1), thereby preventing RAC1-GTP loading without affecting total RAC1 protein abundance (35). These findings confirm that the observed inhibition of proliferation, migration, and invasion (**Figures 6C–E**) is attributable to suppression of RAC1 activation rather than reduction of RAC1 protein expression, further supporting a functional role for active RAC1 signaling in GBM malignant behaviors.

### 3.5 Genetic alteration and immune infiltration analysis of RAC1

**Figure 7:**
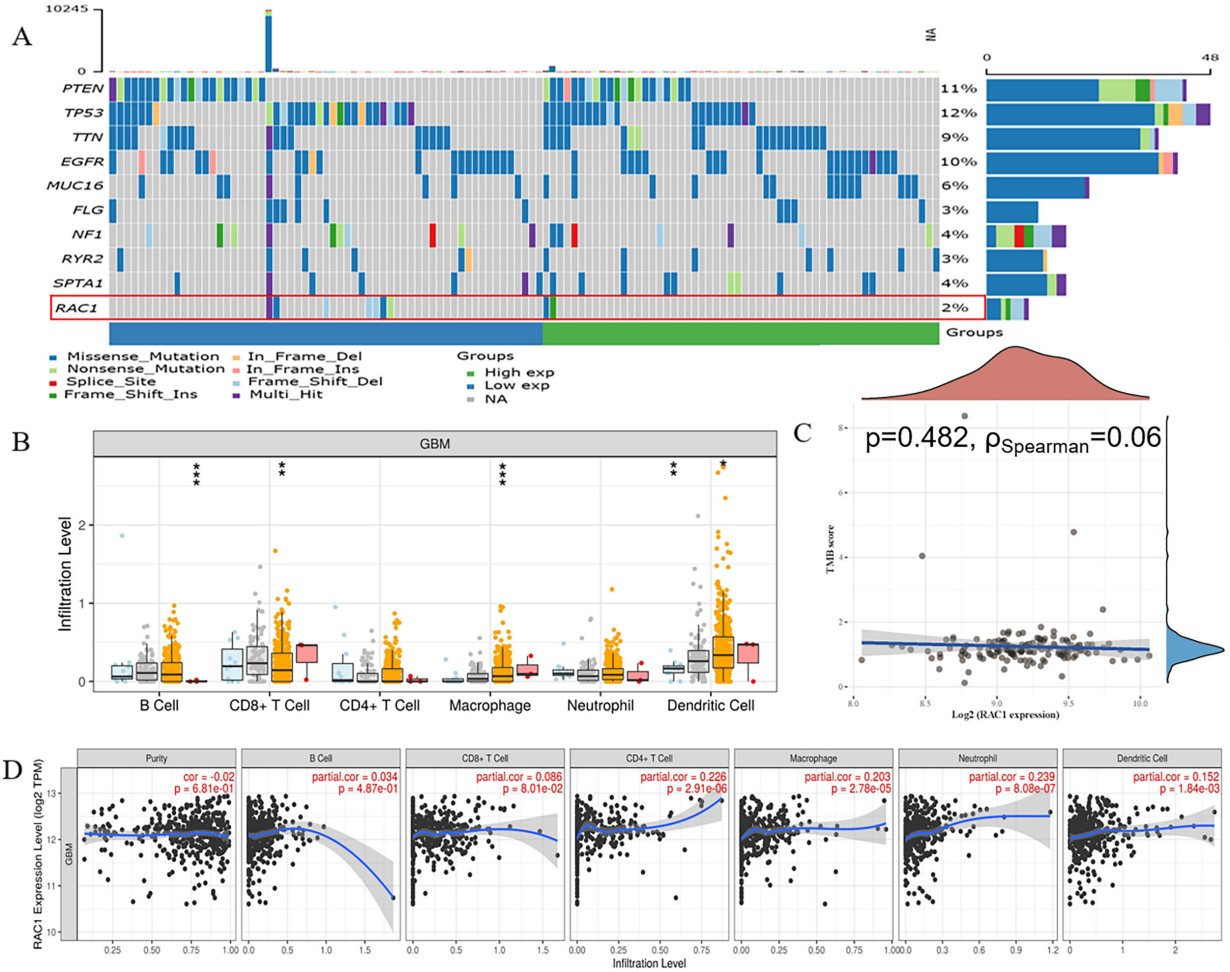
RAC1 mutation and immune cell infiltration. **(A)** Somatic mutation landscape of GBM; **(B)** Relationship between RAC1 mutation and immune cell infiltration in GBM; **(C)** Spearman correlation analysis of TMB and RAC1 gene expression; **(D)** Relationship between RAC1 expression and immune cell infiltration in GBM. (*P < 0.05, **P < 0.01, ***P < 0.001).

Based on TCGA, we analyzed the DNA mutations of RAC1 in various tumors. There is frameshift mutation and insertion in GBM (**Figure 7A**). There was no significant correlation between RAC1 expression and tumor mutation burden (TMB) (**Figure 7C**). We analyzed the correlation between immune cell infiltration and RAC1 expression in GBM (**Figure 7D**). The expression of RAC1 was associated with the infiltration of CD4+ T Cell (Rho=0.226, p=2.91e-06), Macrophage (Rho=0.203, p=2.78e-05), Neutrophil (Rho=0.239, p=8.08e-07), and Dendritic Cell (Rho=0.152, p=1.84e-03). We analyzed the potential relationship between RAC1 mutation and immune cell infiltration in GBM, and found that B Cell, CD8+ T Cell, Macrophage, Dendritic Cell infiltration was related to amplification mutation (**Figure 7B**). Therefore, we believe that the abnormal expression of RAC1 in GBM may be involved in the change of the degree of immune cell infiltration.

### 3.6 Effect of RAC1 expression on responsiveness to immune checkpoint inhibitor therapy

**Figure 8:**
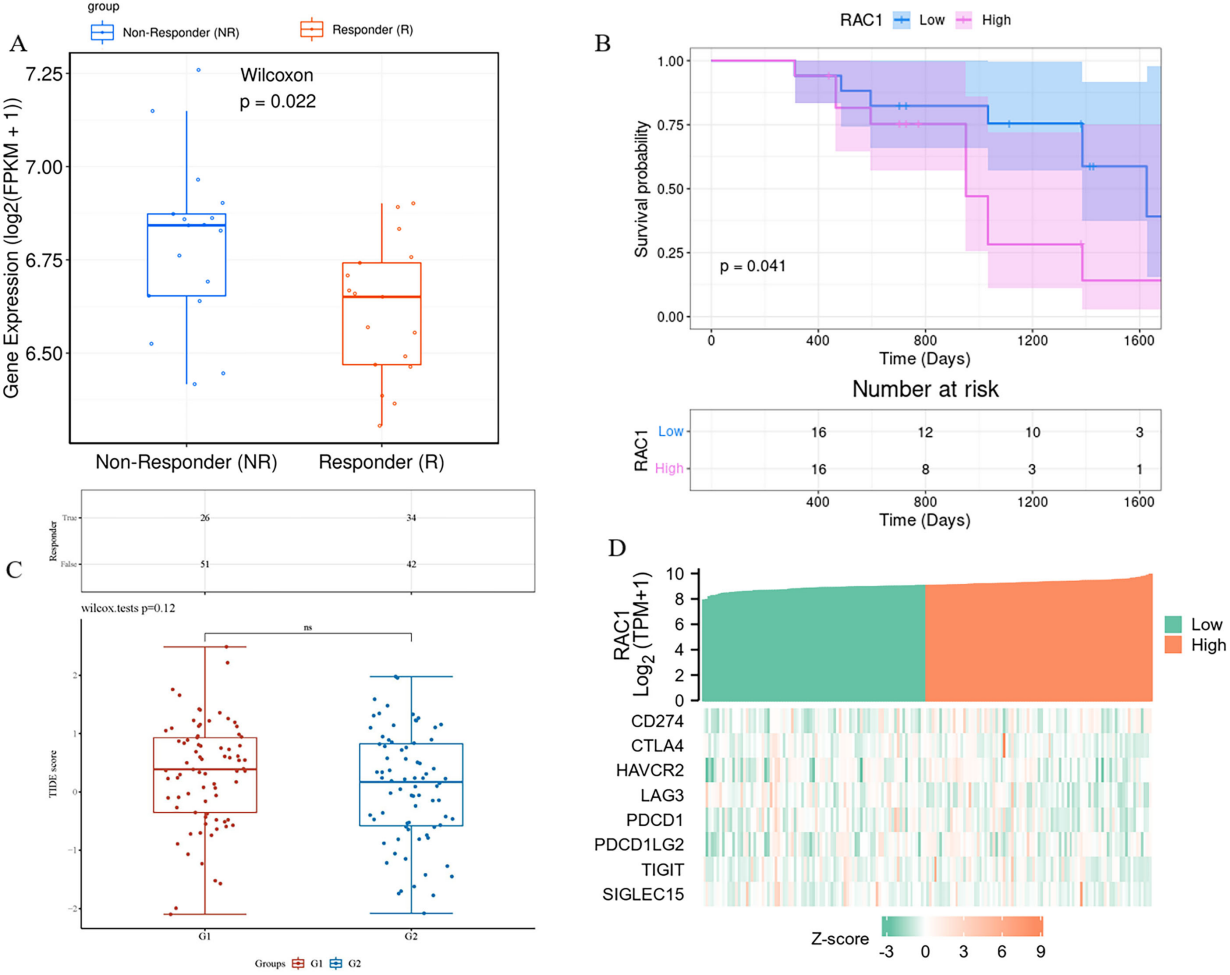
Effect of RAC1 expression on responsiveness to immune checkpoint inhibitor therapy. **(A)** High expression of RAC1 in GBM has a poor response to PD-1 therapy; **(B)** Patients with high RAC1 expression have a poor prognosis in PD-1 therapy; **(C)** TIDE algorithm predicts differential expression of RAC1 does not respond to immune checkpoint inhibitor therapy; **(D)** Correlation heat map between RAC1 expression and immune checkpoint gene expression.

The infiltration of immune cells in tumor microenvironment affects tumor immunotherapy. Immune checkpoint blockade (ICI) is the main method of tumor immunotherapy. At present, the most important drug approved by FDA for ICB treatment is PD-1/PD-L1 inhibitors, but only a limited number of patients respond to PD-1/PD-L1 inhibitors, so it is necessary to screen out predictive biomarkers stratified patients for ICI therapy. Therefore, we further evaluated the immune response to RAC1-related PD-1 therapy based on TIGER. The results showed that RAC1 was highly expressed in the PD-1 treatment resistance group (p=0.04) (**Figure 8A**), and treatment with PD-1 is associated with a poor prognosis if RAC1 expression is high (**Figure 8B**), which suggested that GBM patients with high expression of RAC1 had poor response to PD-1 treatment. We used Tumor Immune Dysfunction and Exclusion (TIDE) algorithm to evaluate the responsiveness. The results showed that the differential expression of RAC1 in GBM patients did not change the response to immune checkpoint inhibitors (**Figure 8C**). The correlation analysis showed that there was no significant correlation between the expression of RAC1 and immune checkpoint genes including CTLA-4 and LAG-3 (**Figure 8D**). The mechanistic basis for these observations is further elucidated by our single-cell analysis (Section 3.9), which demonstrates that PD-L1 (CD274) is essentially absent in GBM tumor cells regardless of RAC1 expression status, while MHC-I antigen presentation components are upregulated in RAC1-high cells, providing a cell-level explanation for why TIDE predicts non-response across both RAC1 expression strata.

### 3.7 Screening of small molecular drugs targeting RAC1 and *in vitro* validation of Palbociclib

**Figure 9:**
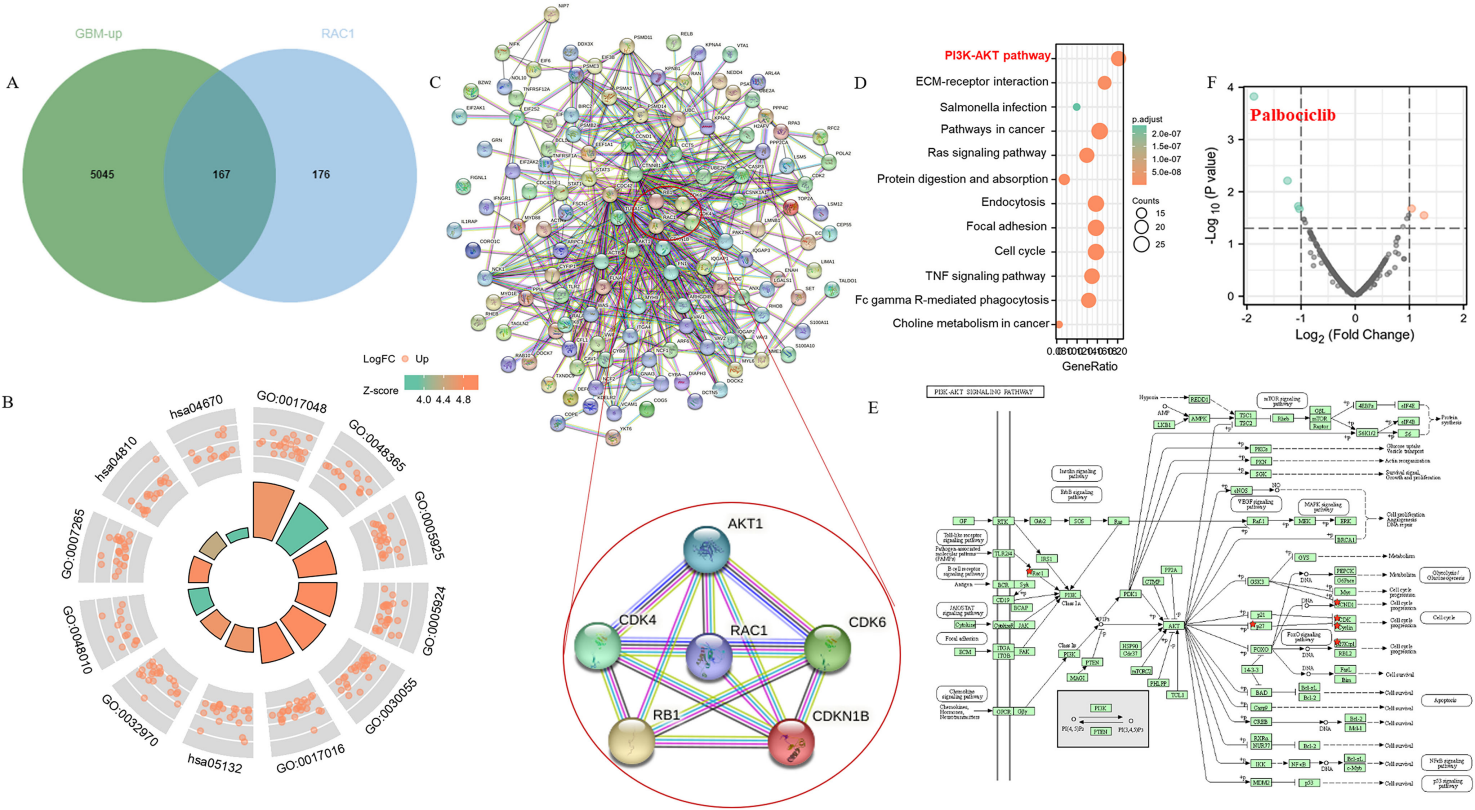
RAC1-related gene enrichment analysis. **(A)** Venn analysis of RAC1 interaction genes and related genes; **(B)** GO enrichment analysis; **(C)** STRING protein interaction network; **(D)** KEGG pathways analysis; **(E)** PI3K-AKT pathway; **(F)** GBM-sensitive small molecular compounds or targeted drugs.

In order to find potential therapeutic targets for stratified GBM patients based on abnormal expression of RAC1, we used GBM differentially expressed genes combined with RAC1 interaction network to find small molecular targeting drugs for GBM patients with high RAC1 expression. We performed enrichment analysis based on RAC1-related differential genes in GBM. There are 343 genes related to RAC1 expression were obtained by STRING and GEPIA, and 5212 highly expressed genes in GBM were screened out by TCGA. Both were subjected to Venn analysis (**Figure 9A**), and 167 genes related to RAC1 and highly expressed in GBM were obtained. The results of GO and KEGG enrichment analysis showed that the main molecular function of these 167 genes was Ras GTPase binding (**Figure 9D**). PI3K-AKT and focal adhesion are mainly involved in their signaling pathways (**Figure 9B**). At the same time, the interaction network map of 167 proteins was constructed based on STRING (**Figure 9C**), and there was a close interaction between RAC1 and RB1, CDK4, CDK6, CDKN1B and AKT1 proteins. Combined with **Figures 9D, E**, it can be seen that RAC1 and CDK4, CDK6 participate in the PI3K-AKT pathway and are related to cell cycle regulation.

**Figure 10:**
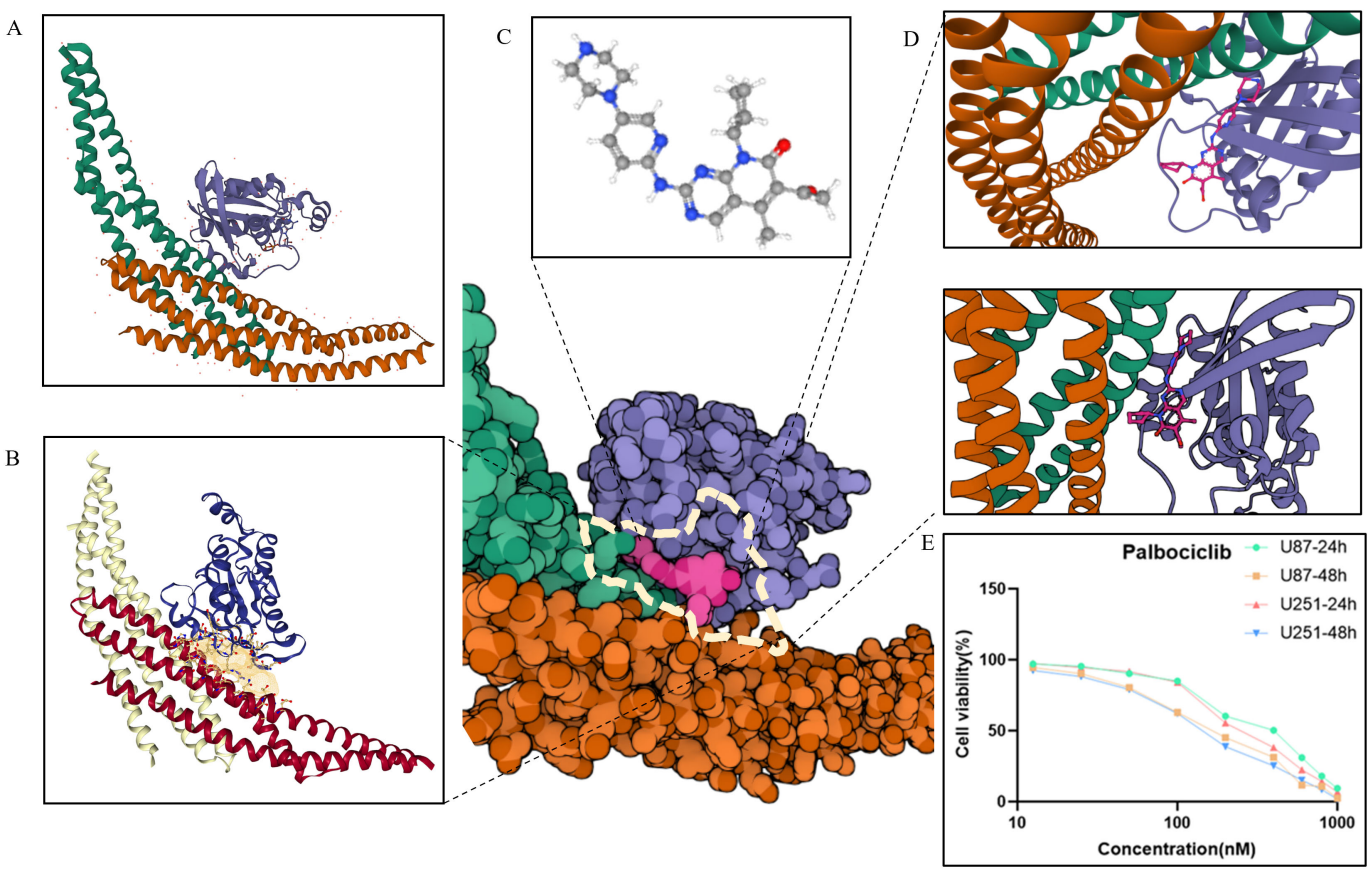
Prediction and validation of RAC1-targeting by Palbociclib. **(A)** The tertiary protein structure of RAC1; **(B)** Prediction of pocket structure of RAC1; **(C)** Molecular structure of Palbociclib; **(D)** Schematic representation of the interaction between RAC1 and Palbociclib; **(E)** CCK-8 cell viability assay of U87 and U251 cells treated with Palbociclib.

We further screened small molecular targeted drugs for the key cycle kinase CDK4/6. Currently, the targeted drugs approved by the FDA to regulate the cell cycle all target the activity of CDK4 and CDK6 proteins, including Palbociclib, Ribociclib, Abemaciclib, Trilaciclib etc. GDSC results showed that Palbociclib (**Figure 9F**) had the highest drug sensitivity in GBM. Molecular docking results showed that RAC1 could bind to Palbociclib (**Figure 10D**), with a binding energy of -7.66 kJ/mol, indicating a predicted binding interaction that warrants further experimental investigation.

To experimentally validate the therapeutic potential of Palbociclib in GBM, we performed CCK-8 cell viability assays on U87 and U251 cells treated with Palbociclib at varying concentrations (**Figure 10E**). The IC50 values were 314.2 nM (24h) and 160.3 nM (48h) for U87 cells, and 257.6 nM (24h) and 142.6 nM (48h) for U251 cells, demonstrating that Palbociclib inhibits GBM cell viability in a concentration- and time-dependent manner. These IC50 values are within the clinically achievable plasma concentration range, supporting the therapeutic relevance of Palbociclib in GBM.

### 3.8 Single-cell transcriptomic analysis reveals RAC1 enrichment in the NPC-like tumor state

**Figure 11:**
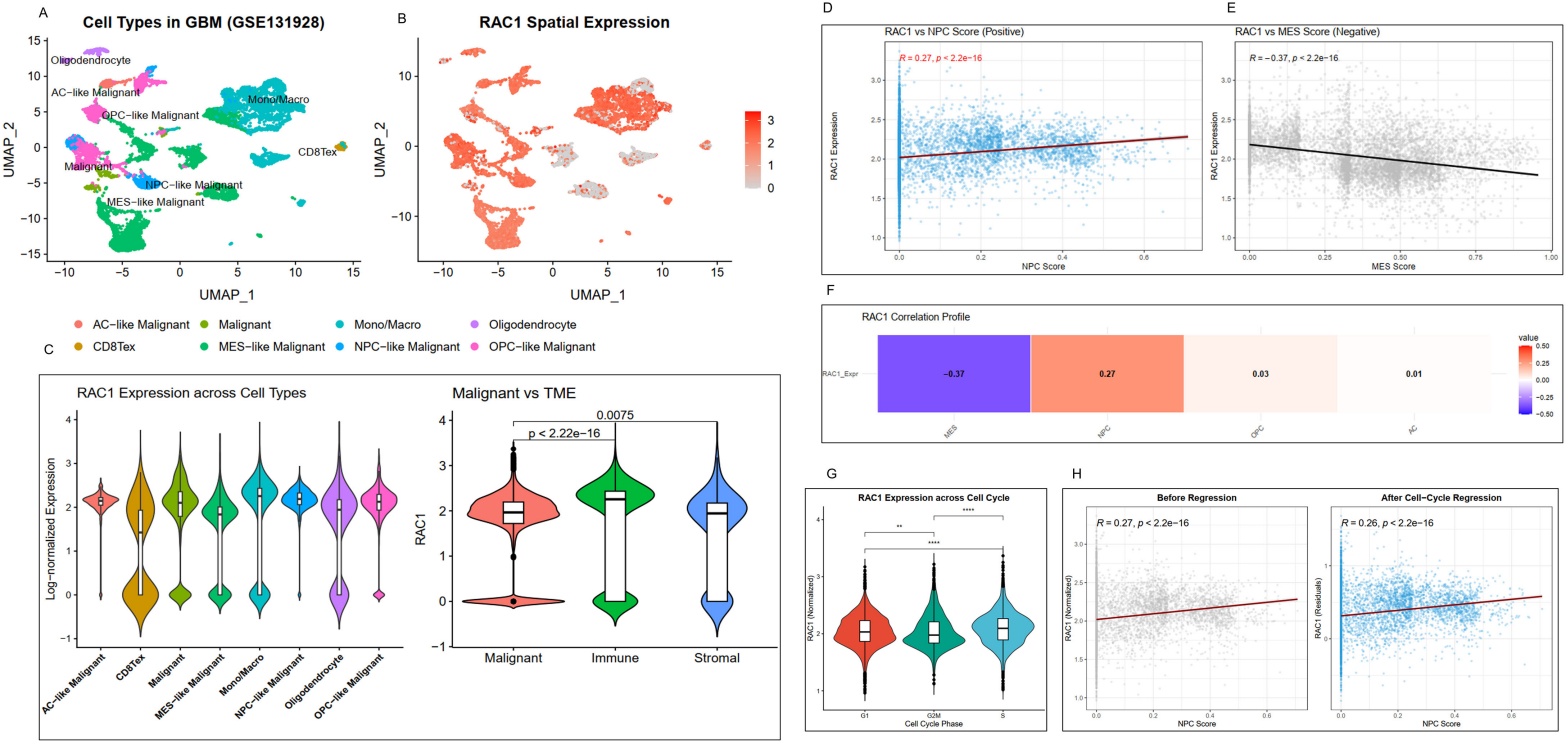
Single-cell analysis of RAC1 expression landscape in GBM. **(A)** UMAP visualization with cell type annotations; **(B)** RAC1 FeaturePlot on UMAP; **(C)** RAC1 expression across cell types **(Violin Plot)** and Malignant vs Immune vs Stromal comparison; **(D)** RAC1 vs NPC state score scatter plot (positive correlation); **(E)** RAC1 vs MES state score scatter plot (negative correlation); **(F)** RAC1 distribution across four tumor states with correlation heatmap; **(G)** RAC1 expression across cell cycle phases (G1 > G2M > S); **(H)** RAC1-NPC correlation before and after cell cycle regression.

To resolve RAC1 expression at single-cell resolution, we analyzed public GBM scRNA-seq data (GSE131928, 10X Chromium dataset). After quality control, batch correction, and clustering, cell types were annotated using annotations from the original publication, identifying malignant cells (AC-like, MES-like, NPC-like, OPC-like), immune populations (Mono/Macro, CD8 Tex), and stromal cells (Oligodendrocyte) (**Figure 11A**). RAC1 was broadly expressed across most cell types, with notably higher expression in immune cells (particularly Mono/Macro) compared to malignant cells, and lowest expression in stromal cells (Malignant vs Immune: p < 2.22e-16; Malignant vs Stromal: p = 0.0075) (**Figures 11B, C**). Within the malignant compartment, AUCell scoring classified tumor cells into four states. RAC1 expression showed a significant positive correlation with NPC-like state score (R = 0.27, p < 2.2e-16) and a significant negative correlation with MES-like state score (R = -0.37, p < 2.2e-16), while showing negligible correlation with OPC-like (R = 0.03) or AC-like (R = 0.01) scores (**Figures 11D–F**).

To determine whether the RAC1-NPC association was merely a by-product of cell proliferation, cell cycle scoring analysis was performed. Unexpectedly, RAC1 expression was highest in G1 phase and lowest in S phase (P < 0.0001; **Figure 11G**), a pattern opposite to canonical proliferation markers such as MKI67. After regressing out S and G2M scores, the correlation between RAC1 and NPC score remained essentially unchanged (R = 0.26, P < 2.2e-16 vs pre-regression R = 0.27; **Figure 11H**), confirming that RAC1 enrichment in the NPC-like state is independent of cell cycle progression. This G1-phase enrichment is consistent with RAC1’s established role in actin cytoskeleton remodeling, a process predominantly active during G1 when cells are competent for migration and fate decisions.

### 3.9 RAC1-high tumor cells exhibit activated signaling modules and PD-L1-independent immune evasion

**Figure 12:**
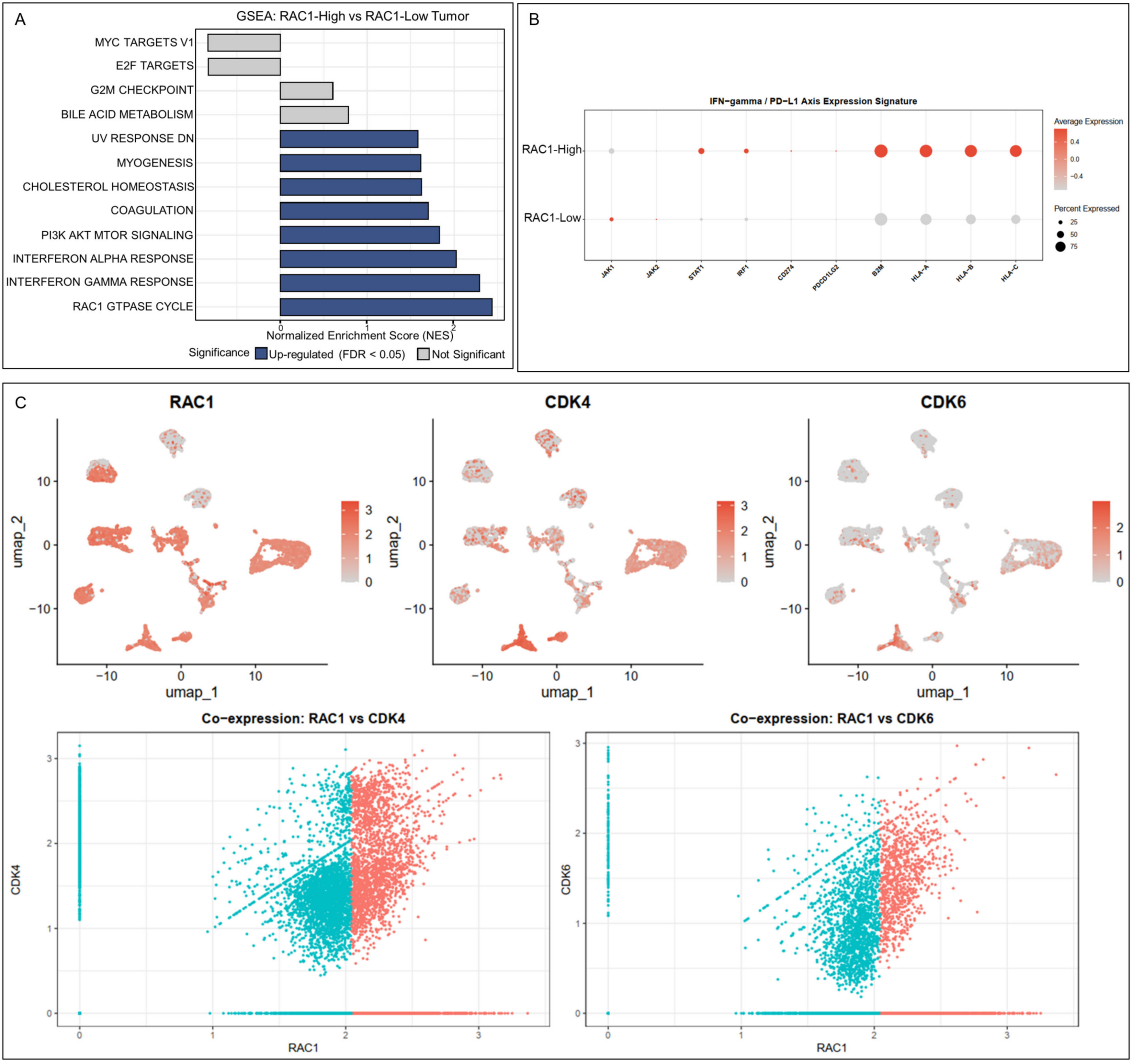
Functional characterization of RAC1-high tumor cells. **(A)** GSEA barplot showing enriched pathways in RAC1-high vs RAC1-low tumor cells (blue, FDR < 0.05; grey, not significant). **(B)** DotPlot of IFN-gamma/PD-L1 axis gene expression in RAC1-high vs RAC1-low tumor cells; dot size indicates percentage of cells expressing the gene, color intensity indicates average expression level. **(C)** FeaturePlot visualization of RAC1, CDK4, and CDK6 expression on the UMAP embedding (upper panels), and single-cell co-expression scatter plots of RAC1 vs CDK4 and RAC1 vs CDK6 (lower panels).

GSEA analysis of RAC1-high versus RAC1-low tumor cells revealed that the RAC1 GTPase Cycle pathway ranked as the top enriched gene set (NES = 2.4, FDR < 0.05; **Figure 12A**), indicating coordinated activation of the entire RAC1 signaling module. Interferon Gamma and Alpha Response pathways were the second and third most enriched (NES = 2.2 and 1.9, respectively), followed by PI3K-AKT-mTOR signaling (NES = 1.7), which cross-validates our bulk-level KEGG pathway analysis (**Figure 9B**). Importantly, canonical proliferation-associated pathways including G2M Checkpoint, E2F Targets, and MYC Targets V1 were not significantly enriched, providing a third line of evidence that RAC1 function in GBM is independent of cell cycle progression (**Figure 12A**).

To dissect the mechanism underlying IFN pathway activation, we examined key IFN-gamma signaling and immune checkpoint molecules using DotPlot analysis (**Figure 12B**). RAC1-high tumor cells showed detectable expression of JAK2, STAT1, and IRF1, which were essentially absent in RAC1-low cells. Downstream, MHC class I antigen presentation components (B2M, HLA-A, HLA-B) were markedly upregulated in RAC1-high cells. However, neither CD274 (PD-L1) nor PDCD1LG2 (PD-L2) showed appreciable expression in either group. This critical finding indicates that the immune evasion mechanism in RAC1-high GBM cells does not operate through the PD-1/PD-L1 axis, consistent with the failure of anti-PD-1 monotherapy in GBM clinical trials and providing a single-cell-level explanation for the TIDE-predicted non-response (**Figure 8C**) and the absence of correlation between RAC1 and checkpoint gene expression (**Figure 8D**).

Single-cell co-expression analysis demonstrated positive correlations between RAC1 and both CDK4 and CDK6 at the individual cell level (**Figure 12C**). FeaturePlot visualization showed that RAC1 and CDK4 exhibited partial spatial co-localization on the UMAP embedding, with both genes enriched in overlapping cell clusters. CDK6 displayed a more dispersed expression pattern with relatively lower overall abundance compared to CDK4, but still showed positive co-expression with RAC1 in a subset of tumor cells. These single-cell co-expression data support the biological rationale for targeting RAC1-high tumors with CDK4/6 inhibitors such as Palbociclib, as both canonical targets of this drug are co-expressed with RAC1 within the same tumor cell populations.

## 4 Discussion

RAC1 is responsible for regulating actin polymerization which impacts cell adhesion, morphology, and movement (36), as well as participate in vesicle transport and cytokinesis (37). Several studies have demonstrated a correlation between abnormal expression of RAC1 and tumor occurrence, progression, or metastasis (9). In order to clarify the abnormal expression of RAC1 in tumors and identify the tumor types with the most predictive potential for RAC1, we comprehensively analyzed the gene expression characteristics of RAC1 in normal tissues and 33 different tumors. We found that RAC1 gene was highly expressed in GBM, LIHC and LUAD, and its high expression was associated with poor prognosis of patients. The ROC curve indicated that the differential expression of RAC1 in GBM had high sensitivity and specificity in predicting the prognosis of patients. Therefore, we speculate that the abnormal expression of RAC1 in GBM may be a potential biomarker to predict the prognosis and treatment response of patients.

Importantly, after identifying GBM as the cancer type with the strongest prognostic relevance for RAC1, we further addressed the question of why RAC1 should be prioritized over other members of the Rho GTPase family. Lasso-Cox regression using all 18 Rho GTPase family members as candidates consistently retained RAC1 across OS, DSS, and PFI endpoints. Subsequent multivariate Cox analysis confirmed RAC1 as the sole family member with an independent hazard ratio exceeding 2 (HR = 2.61, 95% CI: 1.39-4.88, P = 0.003), while other selected genes such as RAC2, RAC3, RHOB, and RND1–3 failed to reach significance. This evidence establishes RAC1’s unique prognostic position within the Rho GTPase family and provides a data-driven rationale for its prioritization in GBM research.

Fine HA et al. observed high expression of RAC1 in glioblastoma tissues by RNA-sequencing of 180 glioblastoma surgical samples (GSE4290). Jung et al. found that overexpressed RAC1 promotes the development of glioblastoma by promoting proliferation and epithelial-stromal transformation in zebrafish model (38). Ou-Yang et al. also found that highly active RAC1 can also work together with phosphorylated AKT to enhance the migration and invasion of glioblastoma (39). Meta-analysis showed that the overexpression of RAC1 was an important index for predicting prognosis (40). Therefore, the abnormal expression of RAC1 in GBM patients may promote the migration and invasion of glioblastoma through different ways, and then affect the prognosis of patients.

A notable observation in our study is the apparent discrepancy between elevated RAC1 mRNA and reduced protein levels observed in CPTAC-GBM and Western blotting (**Figure 6B**). Our single-cell analysis provides insight into this paradox. At single-cell resolution, RAC1 is most highly expressed in immune cells (particularly Mono/Macro), while normal brain tissue used as control in CPTAC and our Western blotting primarily consists of neurons and oligodendrocytes with different RAC1 expression profiles. The difference in cellular composition between tumor tissue (enriched in tumor cells and infiltrating immune cells) and normal brain tissue (enriched in neurons) likely accounts for the apparent protein-level discrepancy when measured in bulk. Furthermore, the CPTAC-GBM phosphoproteomic analysis revealed that total-protein-corrected RAC1 T135 phosphorylation is actually lower in RAC1-high tumors, suggesting that total RAC1 protein levels may not reflect its active GTP-bound form. Importantly, our NSC23766 experiment (**Figure 6F**) provides direct evidence supporting this distinction: NSC23766 inhibited GBM cell proliferation, migration, and invasion without altering total RAC1 protein levels, confirming that RAC1’s oncogenic function depends on its GTP-bound activation state rather than protein abundance. At the molecular level, RAC1 protein is subject to post-translational regulation including ubiquitin-mediated degradation. The E3 ubiquitin ligase HACE1 has been shown to catalyze the polyubiquitylation and subsequent proteasomal degradation of active GTP-bound RAC1 (41), providing a mechanism by which RAC1 protein may undergo rapid turnover despite elevated mRNA transcription in tumor cells. Future studies using RAC1-GTP pull-down assays are warranted to distinguish total protein from active protein levels in GBM.

A key advance of this study is the integration of single-cell transcriptomic analysis, which reveals that RAC1 is preferentially enriched in the NPC-like (neural progenitor cell-like) tumor state as defined by the Neftel classification system (19), rather than the mesenchymal-like state. This enrichment is independent of cell cycle progression, as demonstrated by three converging lines of evidence: (i) RAC1 peaks in G1 phase rather than S/G2M; (ii) cell cycle regression does not alter the RAC1-NPC correlation; and (iii) GSEA shows no enrichment of E2F/MYC/G2M gene sets in RAC1-high cells. The G1-phase enrichment of RAC1 is consistent with its established role in actin cytoskeleton remodeling and lamellipodia formation (42), processes that are predominantly active during G1 when cells are competent for migration and fate decisions. Instead of functioning as a simple proliferation marker, RAC1-high cells exhibit coordinated activation of the entire RAC1 GTPase signaling module (NES = 2.4), PI3K-AKT-mTOR pathway (NES = 1.7), and IFN-gamma response (NES = 2.2). These findings position RAC1 as a marker of an actively signaling, IFN-responsive, proliferation-competent tumor subpopulation. The concurrent enrichment of the RAC1 GTPase cycle as the top-ranked pathway further indicates that RAC1’s prognostic impact reflects the activation of an entire signaling network rather than isolated gene overexpression, consistent with the known function of RAC1 as a molecular switch that coordinates multiple downstream effectors including PAK1 and the WAVE-ARP2/3 complex (3, 42).

Immune microenvironment played a key role in tumor biology. Preclinical and clinical immunotherapy have been achieved for many tumor types. However, the role of immunotherapy in glioma remains to be elucidated (43, 44). A critical process in immunotherapy is to determine which types of tumor patients could benefit from immune checkpoint inhibitors (ICIs). On this basis, we conducted an in-depth analysis about the possibility of RAC1 as a marker for predicting immunotherapy responsiveness. We found that high expression of RAC1 in GBM was associated with the infiltration of various immune cells but not TMB. Furthermore, the analysis of immunotherapy responsiveness showed that GBM patients with high RAC1 expression were resistant to PD-1 therapy and might also be unresponsive to other ICI treatments. The correlation analysis of immune checkpoint gene expression and RAC1 expression also confirmed the result of high RAC1 expression on PD-1 treatment resistance. Studies suggest that tumors with high TMB often carry higher levels of neoantigens, which may lead to the activation of T cells, thus improving the therapeutic sensitivity of ICIs (18, 45, 46). Cao et al. conducted a collective analysis on a total of 103,078 patients to assess the predictive accuracy of TMB. Nevertheless, they discovered that the predictive capability of TMB varied significantly among patients who received ICIs, and this variability may be linked to the types of tumors (47). A multinational clinical trial in 2022 confirmed that GBM was considered as “immunologically cold tumors” and did not respond to ICI (18). This may be related to immune checkpoint blockade caused by low TMB and extensive intra-tumor heterogeneity in GBM. Hence, to utilize TMB in practical applications, it is imperative to establish and assess its precise function in various tumors through further clinical trials. This poses a unique challenge to targeted immunotherapy.

Our single-cell analysis provides a mechanistic explanation for these bulk-level observations. At single-cell resolution, we demonstrate that PD-L1 (CD274) is essentially absent from GBM tumor cells regardless of RAC1 status, while RAC1-high tumor cells exhibit active IFN-gamma signaling (JAK2, STAT1, IRF1) and upregulated MHC-I antigen presentation (B2M, HLA-A/B). This creates a paradoxical phenotype: RAC1-high tumor cells are theoretically visible to cytotoxic T cells yet evade immunity through PD-L1-independent mechanisms. This finding is consistent with the failure of anti-PD-1 monotherapy in multiple GBM clinical trials, including CheckMate 143, which demonstrated that nivolumab did not improve overall survival over bevacizumab in recurrent GBM (48), and CheckMate 498, which showed no benefit of nivolumab combined with radiotherapy over temozolomide combined with radiotherapy in newly diagnosed MGMT-unmethylated GBM (49). The absence of PD-L1 expression at the single-cell level provides a direct molecular explanation for these clinical failures and for the TIDE-predicted non-response observed in our bulk-level analysis (**Figure 8C**): the PD-1/PD-L1 axis is simply not the operative immune checkpoint in GBM tumor cells.

The alternative immune evasion mechanisms in RAC1-high GBM warrant further investigation. Our bulk-level analysis showed that RAC1 expression was positively correlated with macrophage infiltration (Rho = 0.203, P = 2.78e-05; **Figure 7D**), suggesting that TAM-mediated immunosuppression may constitute a major immune barrier in RAC1-high tumors. Pyonteck et al. have demonstrated that CSF-1R-dependent macrophage reprogramming can alter glioma progression and microenvironment composition in preclinical models (50). Additionally, immunosuppressive metabolic pathways such as IDO1-mediated tryptophan catabolism have been implicated in GBM immune evasion; Wainwright et al. showed that IDO expression in brain tumors increases regulatory T cell recruitment and negatively impacts patient survival (51). Whether RAC1-high tumor cells preferentially engage these non-PD-L1 escape routes remains to be elucidated in future studies, but the convergence of active IFN-gamma signaling, intact MHC-I, absent PD-L1, and elevated macrophage infiltration strongly suggests that the immune barrier in RAC1-high GBM is primarily microenvironment-mediated rather than tumor-intrinsic checkpoint-driven. The clinical implication is that RAC1 as a stratification biomarker should guide patients toward alternative therapeutic strategies rather than ICI monotherapy.

Since the differential expression of RAC1 in GBM patients has no significant effect on the response of immune checkpoint inhibitors, it is not clear whether the high expression of RAC1 in GBM patients indicates better clinical benefits of CDK4/6 targeted inhibitors therapy. In this study, we found that RAC1 has a close interaction with the cell cycle regulatory protein CDK4/6 and jointly participates in the PI3K-AKT pathway, which is then related to cell cycle regulation. Our single-cell analysis provides additional support: RAC1 and CDK4 show positive co-expression at the individual cell level, with spatial co-localization in NPC-like tumor cells. Furthermore, RAC1 expression peaks in G1 phase, precisely the cell cycle stage where Palbociclib exerts its therapeutic effect by blocking the G1-to-S transition. GDSC database results showed that Palbociclib had high drug sensitivity to GBM, molecular docking predicted a binding interaction between RAC1 and Palbociclib (binding energy -7.66 kJ/mol), and importantly, our *in vitro* CCK-8 assays confirmed that Palbociclib inhibits U87 and U251 cell viability with IC50 values in the nanomolar range (142.6-314.2 nM), well within the clinically achievable plasma concentration. CDK4/6 inhibitors play an anti-tumor role by inhibiting the activity of CDK4/6, blocking the progress of cells from G1 phase to S phase (52). Other clinical trials, including one for retinoblastoma proficient glioblastoma (Clintrial.gov identifier: NCT01227434), are also under way. Michaud et al. demonstrated the potential efficacy of palbociclib in a preclinical orthotopic GBM mouse model (53, 54). Yu et al. (55) demonstrated that Palbociclib inhibits the survival of GBM xenografts. Further studies revealed that in U251 and LN229 glioma cells, Palbociclib suppressed cell proliferation and migration by downregulating the overexpression of TCF12 and VSIG4 (56). Similarly, He et al. (57) found that Palbociclib/BIX-02189 reduced the expression of GINS2 and inhibited glioma cell proliferation. These findings, together with our computational and experimental evidence, support Palbociclib as a candidate therapeutic agent for RAC1-high GBM patients.

Integrating our bulk and single-cell findings, we propose a therapeutic stratification framework for GBM patients based on RAC1 expression. RAC1-high patients harbor NPC-like tumor populations with active PI3K-AKT signaling and CDK4 co-expression, suggesting potential benefit from CDK4/6 inhibitor-based strategies. Simultaneously, the active IFN-gamma signaling and intact MHC-I expression in RAC1-high cells suggest that these tumors possess latent immunogenicity that could be unlocked by removing non-PD-L1 immune barriers. Given the strong correlation between RAC1 expression and macrophage infiltration observed in our data, combination of CDK4/6 inhibitors with TAM-targeted agents such as CSF-1R inhibitors (50) may represent a particularly rational approach for RAC1-high GBM patients. This combined strategy addresses both the tumor-intrinsic proliferative driver (via CDK4/6 inhibition) and the microenvironment-mediated immune barrier (via TAM reprogramming) simultaneously, and warrants future clinical investigation.

To sum up, we first analyzed the expression and survival of RAC1 in various tumors, among which RAC1 had the highest accuracy in predicting the prognosis of patients with GBM. The selection of RAC1 was then validated through unbiased Lasso-Cox regression and multivariate Cox analysis across the entire Rho GTPase family, confirming RAC1 as the sole independent prognostic risk factor. On this basis, the correlation between the abnormal expression of RAC1 in GBM and immune cell infiltration and TMB was analyzed, and it was found that the stratification of GBM patients based on RAC1 expression can be used as an index to evaluate whether they benefit from immunotherapy. Single-cell analysis further revealed that RAC1 marks an NPC-like tumor state with active IFN-gamma signaling but PD-L1-independent immune evasion, providing a mechanistic explanation for ICI resistance. At the same time, the small molecular drug Palbociclib which may bind to RAC1 was screened by enrichment analysis and molecular docking, and validated by *in vitro* CCK-8 assays, providing a new therapeutic strategy for GBM.

However, there are still some limitations in our research. Although data sets such as TCGA, GTEx and GEO are included, the number of samples is still limited. Second, given the individual differences in cancer patients, it is difficult for the study to cover all possible differences. Third, while molecular docking predicted a binding interaction between Palbociclib and RAC1, and CCK-8 assays confirmed Palbociclib’s anti-proliferative effect on GBM cells, direct evidence for Palbociclib specifically targeting RAC1 protein (as opposed to its canonical CDK4/6 targets) requires further validation through RAC1-specific knockdown/overexpression experiments combined with Palbociclib treatment. Fourth, our single-cell analysis was performed on a single public dataset (GSE131928); validation in additional scRNA-seq cohorts or spatial transcriptomics data would further strengthen the findings. Finally, this study provides some potential treatments related to RAC1 based on public database and bioinformatics screening, but it is necessary to further verify the molecular mechanism by which RAC1 gene signature affects GBM prognosis at the molecular level *in vitro*.

## Data Availability

The original contributions presented in the study are included in the article/**Supplementary Material**. Further inquiries can be directed to the corresponding authors.
