# Supplementary material for: Targeting RAC1 in glioblastoma: prognostic value, immune landscape, and small molecule therapeutic potential
Source: Front Oncol. 2026 Jun 18;16:1801747. doi: 10.3389/fonc.2026.1801747 (PMC13322918; doi:10.3389/fonc.2026.1801747)
Supplement: Supplementary file 1 [file Table1.docx]

**Targeting RAC1 in Glioblastoma: Prognostic Value, Immune Landscape, and Small Molecule Therapeutic Potential**

**Author names and Affiliations:**

Qionghui Wu^a#^, Gang Su^b#^, Xiaodong Xie^b^, Shanlin Chen^c^, Xinli Feng^a^, Zhenchang Zhang^a^*

a Lanzhou University Second Hospital, Lanzhou University, Lanzhou 730000, Gansu Province, China;

b Institute of Genetics, School of Basic Medical Sciences, Lanzhou University, Lanzhou 730000, Gansu Province, China;

c Department of Neurosurgery, Liangzhou Hospital of Wuwei, Wuwei 733000, Gansu Province, China;

*Corresponding author: Zhenchang Zhang (Email: [tougao13893647595@163.com](mailto:tougao13893647595@163.com))

**The valid institutional email address for each author:**

Qionghui Wu: 120220901840@lzu.edu.cn

Gang Su: sugang@lzu.edu.cn

Shanlin Chen: 85621937@qq.com

Xiaodong Xie: xdxie@lzu.edu.cn

Xinli Feng: [220220905340@lzu.edu.cn](mailto:220220905340@lzu.edu.cn)

**Journal name:**

**Molecular Biotechnology**

Supplement Table 1. Tools and database mentioned in materials and methods

| Abbreviation | Full Name | Version | Website Address |
| --- | --- | --- | --- |
| TCGA | The Cancer Genome Atlas | 29.0 | https://portal.gdc.cancer.gov/ |
| TIMER2 | Tumor immune estimation resource | 2.0 | http://timer.cistrome.org/ |
| GEPIA | Gene expression profiling interactive analysis | 2.0 | http://gepia2.cancer-pku.cn/ |
| GTEx | Genotype-Tissue Expression | / | https://www.genome.gov/Funded-Programs-Projects/Genotype-Tissue-Expression-Project |
| UALCAN | / | 2021-03-06 | http://ualcan.path.uab.edu/analysis-prot.html |
| HPA | Human Protein Atlas | 20.1 | http://www.proteinatlas.org/ |
| KM plotter | Kaplan-Meier Plotter | 2021.08.04 | https://kmplot.com/analysis/ |
| cBioPortal | / | 3.6.20 | https://www.cbioportal.org/ |
| STRING | Protein-Protein Interaction Networks Functional Enrichment Analysis | 11.0 | https://string-db.org/ |
| Jvenn | / | / | http://jvenn.toulouse.inra.fr/app/example.html |
| Metascape | / | 3.5 | https://metascape.org/gp/index.html#/main/step1 |
| TIGER | Tumor Immunotherapy Gene Expression Resource | / | http://tiger.canceromics.org/ |
| PubChem | / | / | https://pubchem.ncbi.nlm.nih.gov/ |
| GDSC | Genomics of Drug Sensitivity in Cancer | / | https://www.cancerrxgene.org/ |
| PDB | Protein Data Bank | / | http://www.rcsb.org/pdb/home/home.do |
| Autodock Vina | / | 1.2.2 | http://autodock.scripps.edu/ |
| The R Project | The R Programming Language | v4.0.3 | https://www.r-project.org/ |
| TIDE | Tumor Immune Dysfunction and Exclusion |  | http://tide.dfci.harvard.edu/ |

Supplement Table 2. Abbreviation of tumors mentioned in results

| Tumor name | Abbreviation |
| --- | --- |
| bladder urothelial carcinoma | BLCA |
| cholangio carcinoma | CHOL |
| esophageal carcinoma | ESCA |
| glioblastoma multiforme | GBM |
| head and neck squamous cell carcinoma | HNSC |
| head and neck squamous cell carcinoma- HPV+ | HNSC-HPV+ |
| kidney chromophobe | KICH |
| kidney renal clear cell carcinoma | KIRC |
| kidney renal clear cell carcinoma | KIRP |
| liver hepatocellular carcinoma | LIHC |
| lung adenocarcinoma | LUAD |
| lung squamous cell carcinoma | LUSC |
| prostate adenocarcinoma | PRAD |
| stomach adenocarcinoma | STAD |
| thyroid carcinoma | THCA |
| uterine corpus endometrial carcinoma | UCEC |
| adrenocortical carcinoma | ACC |
| lymphoid neoplasm diffuse large B-cell lymphoma | DLBC |
| acute myeloid leukemia | LAML |
| brain lower grade glioma | LGG |
| ovarian serous cystadenocarcinoma | OV |
| sarcoma | SARC |
| testicular germ cell tumors | TGCT |
| thymoma | THYM |
| uterine carcinosarcoma | UCS |
| pancreatic adenocarcinoma | PAAD |
| testicular germ cell tumor | TGCT |
| mesothelioma | MESO |
| colon adenocarcinoma | COAD |
| skin cutaneous melanoma | SKCM |
| breast invasive carcinoma | BRCA |
| cervical squamous cell carcinoma and endocervical adenocarcinoma | CESC |
