# Supplementary figures and images for: Targeting RAC1 in glioblastoma: prognostic value, immune landscape, and small molecule therapeutic potential
Source: Front Oncol. 2026 Jun 18;16:1801747. doi: 10.3389/fonc.2026.1801747 (PMC13322918; doi:10.3389/fonc.2026.1801747)

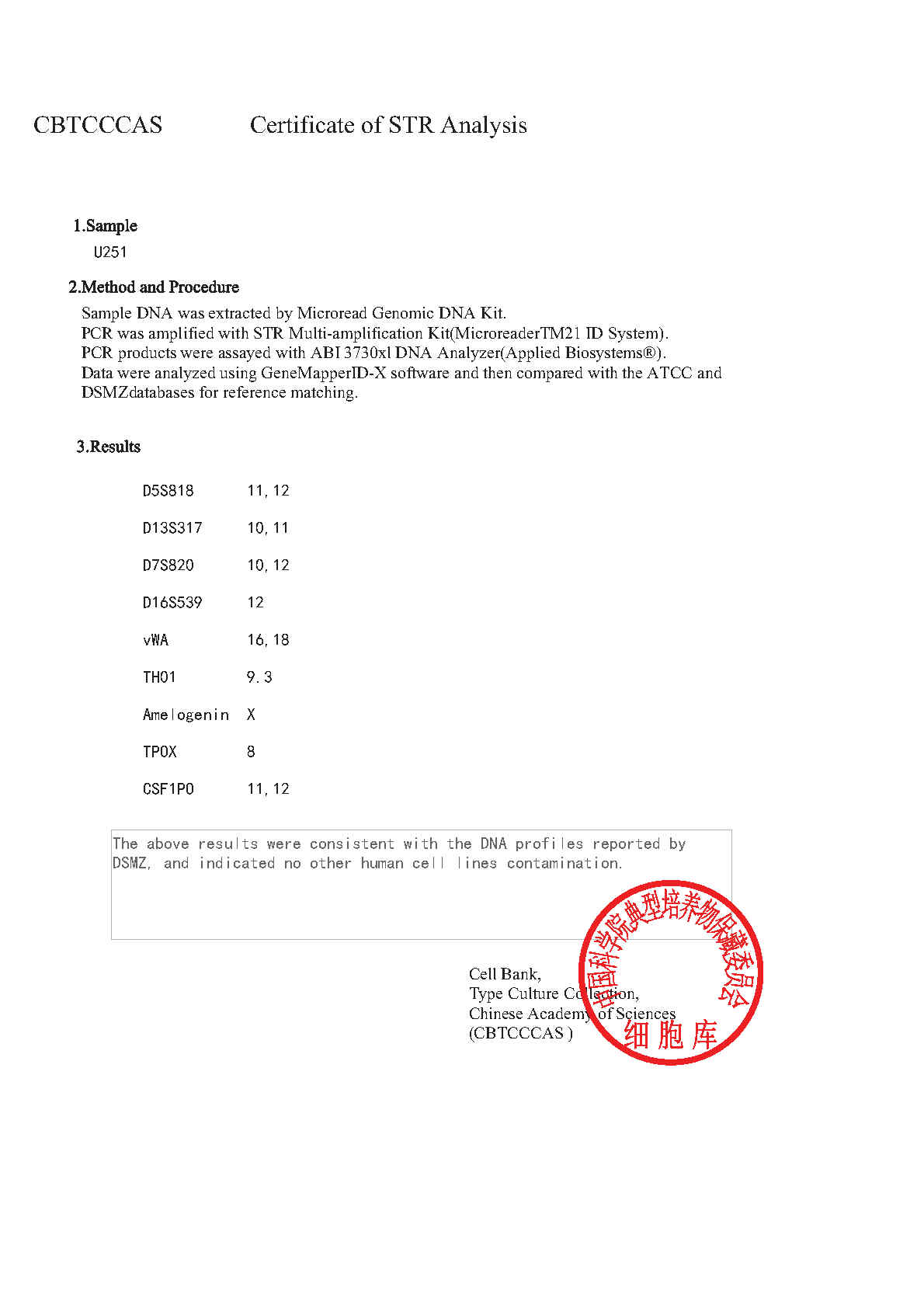

Supplement: Supplementary file 2 [file Image1.jpg]
